# Meal‐Induced Proton Density Fat Fraction and ${{\text{T}}_{2}}^{*}$ Decrease in Supraclavicular Adipose Tissue

**DOI:** 10.1002/nbm.70414

**Published:** 2026-10-05

**Authors:** Johannes Raspe, Tianxing Du, Mingming Wu, Jonathan Stelter, Martin Klingenspor, Daniela Junker, Thomas Skurk, Dimitrios C. Karampinos

**Affiliations:** ^1^ Institute for Diagnostic and Interventional Radiology, School of Medicine and Health TUM University Hospital, Technical University of Munich (TUM) Munich Germany; ^2^ ZIEL – Institute for Food & Health, Core Facility Human Studies Technical University of Munich (TUM) Freising Germany; ^3^ Department of Radiology LMU University Hospital Munich Germany; ^4^ Laboratory of Magnetic Resonance Imaging Systems and Methods, École Polytechnique Fédérale de Lausanne (EPFL) Lausanne Switzerland; ^5^ EKFZ – Else Kröner‐Fresenius Center for Nutritional Medicine Technical University of Munich (TUM) Freising Germany; ^6^ Chair of Molecular Nutritional Medicine, School of Life Sciences Technical University of Munich (TUM) Freising Germany; ^7^ School of Medicine and Health TUM University Hospital, Technical University of Munich (TUM) Munich Germany; ^8^ CIBM Center for Biomedical Imaging (CIBM) Lausanne Switzerland

**Keywords:** brown adipose tissue (BAT), meal activation, proton density fat fraction (PDFF) mapping, ${{\text{T}}_{2}}^{*}$

## Abstract

Brown adipose tissue (BAT) is a metabolically active tissue in humans, located primarily within the supraclavicular adipose tissue (scAT), that can be activated by cold or high‐caloric meal consumption. While the changes of proton density fat fraction (PDFF) upon cold activation are well investigated, there is a knowledge gap regarding PDFF and ${{\text{T}}_{2}}^{*}$ dynamics upon meal‐induced BAT activation. Therefore, the purpose of this study was to establish a robust pipeline to quantify PDFF and ${{\text{T}}_{2}}^{*}$ changes in scAT in fasting control and meal intervention experiments. To that end, a motion‐robust radial stack‐of‐stars trajectory has been employed with a regularized iterative reconstruction algorithm, graph‐cut‐based water‐fat separation, affine image registration and automatic neural‐network‐based region of interest (ROI) segmentation. Next to scAT, ROIs of subcutaneous adipose tissue (SAT) and muscle have been created to compare the results to reference tissues. Multi‐echo gradient echo acquisitions were repeated in a total of ten volunteers before a break, in which the volunteers consumed an iso‐caloric meal in the meal intervention experiment. After the break, repeated multi‐echo gradient echo measurements were used to dynamically track the changes of both PDFF and ${{\text{T}}_{2}}^{*}$. In the meal intervention experiment, scAT PDFF showed a stronger decrease of $∼1{\%}_{\mathrm{PDFF}}$ compared to the fasting control experiment, in which the decrease below baseline was similar to that in SAT. Furthermore, scAT ${{\text{T}}_{2}}^{*}$ exhibited a strong decrease to −3.2ms below baseline after meal intervention, while no decrease was observed in the fasting control experiment or in SAT. Considering the observed dynamic effects and their differences to the fasting control experiment, a PDFF decrease of $∼1{\%}_{\mathrm{PDFF}}$ and a ${{\text{T}}_{2}}^{*}$ decrease of ∼3ms could be attributed to meal‐induced activation of scAT.

## Graphical abstract

In two session per volunteer–fasting control and meal activation–, the volunteers underwent repeated multi‐echo gradient echo acquisitions, which were subsequently processed offline with reconstruction of the source images, water‐fat separation with ${T_{2}}^{*}$ mapping, affine registration, and automated segmentation of three regions of interest. The supraclavicular region showed the strongest decrease of proton density fat fraction (PDFF) and ${T_{2}}^{*}$ in line with the expected behavior of brown adipose tissue (BAT) activation, while the control regions showed none or little change. Since PDFF decreased also under fasting control conditions, ${T_{2}}^{*}$ was found to be the seemingly more sensitive parameter to track postprandial dynamics in suspected BAT.

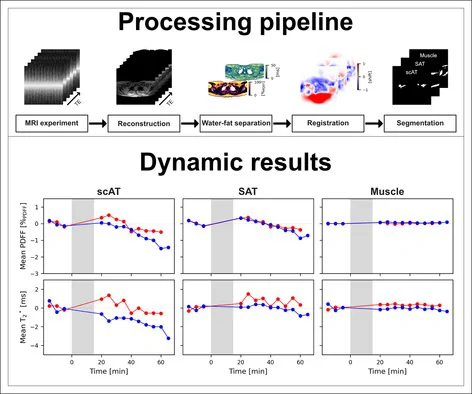

## 1 Introduction

Brown adipose tissue (BAT) is a specialized thermogenic adipose organ that dissipates energy as heat through sympathetic activation of uncoupling protein 1 (UCP1) in brown adipocytes (BAs) [1]. Relative to white adipose tissue (WAT), BAT is characterized by smaller lipid droplets, dense vascularization and high mitochondrial content, features that support rapid substrate mobilization, heat production and dissipation [1]. In adult humans, BAs are predominantly found as clusters within distinct depots, with the largest contiguous region located within the supraclavicular adipose tissue (scAT) [2, 3, 4]. As a recruitable thermogenic tissue, BAT is classically activated by cold exposure, however, meal intake has also been implicated as a physiological trigger of BAT activity, raising the question of how postprandial BAT responses can be quantified in vivo [5, 6, 7]. Given its emerging relevance for inter‐individual differences in energy metabolism and cardiometabolic risk, BAT is increasingly considered a potential target and biomarker for precision medicine approaches in obesity and metabolic disease [8].

Given its important role in the energy metabolism, BAT and its response to external stimuli have been studied since its rediscovery in adult humans via positron emission tomography (PET) with computed tomography (CT) in several studies, characterizing the distribution and activation of human BAT in vivo [2, 3, 9]. Different tracers have been employed, including 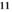
C‐acetate [10], 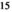
O [11] and 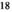
F‐fluorodeoxyglucose (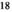
F‐FDG) [2, 4]. Performing studies employing PET/CT with the most widely used tracer 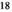
F‐FDG enables the assessment of glucose uptake as a surrogate marker of metabolic and thermogenic activity but has some limitations. 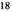
F‐FDG PET/CT does not directly measure thermogenesis, but rather reflects glucose utilization [12], which only represents one aspect of BAT metabolism. This caveat is answered by using 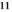
C‐acetate or 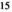
O as tracer, that directly probe the metabolism, but require more complex infrastructure including an on‐site cyclotron. Another caveat affecting PET/CT independent of the tracer used is the use of ionizing radiation from CT and the radioactive tracer from PET. This limits its applicability, particularly in longitudinal or repeated‐measurement studies in healthy or young populations. Therefore, MRI has been used as a radiation‐free alternative imaging methodology to characterize BAT under activation, mostly to assess changes in the proton density fat fraction (PDFF) and ${{\text{T}}_{2}}^{*}$ of scAT [13, 14].

Chemical shift‐encoding (CSE)‐based water‐fat separation (WFS) MRI allows simultaneous mapping of both parameters, PDFF, and ${{\text{T}}_{2}}^{*}$, in adipose tissue using multi‐echo gradient echo acquisitions [15]. PDFF reflects the ratio of mobile fat protons to mobile protons of fat and water and serves as a widely used quantitative measure of tissue fat content [16]. In scAT, cold‐induced BAT activation is expected to mobilize intracellular triglycerides to fuel thermogenesis [10], which would decrease local lipid content and thus reduce PDFF [17]. Indeed, several human MRI studies have reported lower PDFF in scAT compared to pure WAT regions [13, 14, 18], and dynamic decreases in scAT PDFF during or after cold exposure have been interpreted as markers of BAT lipid utilization [14]. However, BAT metabolism is complex, and several competing processes may influence PDFF. For instance, BAs with low initial lipid content may increase their lipid storage during lipogenesis, whereas lipolysis reduces intracellular triglyceride content [19, 20]. At the same time, increased blood volume and vascular dilation raise the water signal and can therefore reduce the apparent fat fraction independent of changes in lipid content [21]. As a result, BAT activation may lead to either increases or decreases in PDFF, depending on the relative contributions of lipid turnover and water‐related effects. In parallel, BAT activation is associated with increased mitochondrial activity and changes in vascular volume and oxygenation [19], which alter the local iron content and magnetic environment. Elevated concentrations of paramagnetic species, such as iron in mitochondria or deoxyhemoglobin, and microscopic susceptibility differences shorten ${{\text{T}}_{2}}^{*}$, so a decrease in ${{\text{T}}_{2}}^{*}$ has been proposed as a potential MR marker of BAT activation [13, 22].

Building on these proposed MRI markers, several studies have investigated the effects of a cold stimulus on MRI‐derived parameters of scAT, some combining MRI with 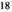
F‐FDG PET/CT [23, 24, 25, 26, 27], using PET/MRI [13, 28, 29] or PET with magnetic resonance spectroscopy [30] to probe lipid composition. In many of those studies, PDFF was dynamically tracked over time [17, 22, 31, 32, 33], most of them report a decrease in PDFF following cold exposure [17, 31, 32, 33] consistent with BAT activation, while only few studies additionally assessed ${{\text{T}}_{2}}^{*}$, reporting mixed results [22, 32]. Against this background, although PET/CT has provided evidence that a high‐caloric meal can activate human BAT [6, 11], systematic MRI studies characterizing dynamic PDFF and ${{\text{T}}_{2}}^{*}$ changes in scAT after meal‐induced BAT activation in humans are still lacking. This knowledge gap is particularly interesting to fill, because postprandial BAT activation is directly relevant to everyday energy balance and metabolic health [34], and MRI allows repeated, radiation‐free measurements in the same individuals. However, quantification of ${{\text{T}}_{2}}^{*}$ in scAT is technically particularly challenging due to its sensitivity to motion, $B_{0}$ inhomogeneities and susceptibility effects [35, 36], which can vary across the neck. Recent work found significantly improved ${{\text{T}}_{2}}^{*}$ quantification in scAT using a radial stack‐of‐stars (SoS) acquisition, which is more robust to motion compared to conventional Cartesian acquisitions and enables retrospective correction of motion‐induced spatially varying $B_{0}$ inhomogeneities that have a large effect on ${{\text{T}}_{2}}^{*}$ quantification [37]. Such an acquisition technique is, therefore, particularly suited for dynamic studies, where subtle time‐dependent changes need to be detected reliably.

Next to technical challenges during the acquisition, there are other issues potentially limiting the reliability and reproducibility of previously conducted studies. The reconstruction and processing including WFS have often relied on the vendor implementation and are, therefore, not under the control of the operator in case of water‐fat swaps occurring. Furthermore, both PDFF and ${{\text{T}}_{2}}^{*}$ quantification was often impaired by a limited amount of echoes, sometimes relying on two‐point Dixon‐type scans. The regularly employed Cartesian acquisition is particularly susceptible to motion and fluctuating $B_{0}$ inhomogeneities [35]. Lastly, the ROIs for analysis and quantification were mostly manually generated and varied across studies. However, manual segmentation of the complex anatomy of scAT was shown to be of limited inter‐observer agreement [38, 39].

The aim of the present study was to investigate meal‐induced changes in MRI‐derived parameters associated with BAT activation, namely PDFF and ${{\text{T}}_{2}}^{*}$, in human scAT while addressing potential limitations from prior activation studies. Thereby, this study utilized the tomographic nature of MRI which allows to investigate these parameters in three‐dimensional volumes, enabling spatially resolved assessment of meal‐induced changes within scAT and in white adipose reference tissues. For that, a reproducible pipeline was introduced, starting from a robust radial SoS acquisition and incorporating iterative reconstruction, swap‐free WFS, affine registration, and automatic tissue segmentation using established neural network‐based tools, ensuring consistent quantification across subjects and time points.

## 2 Materials and Methods

### 2.1 Study Design

The study employed two visits per volunteer, both visits consisting of a standardized dinner and overnight fasting as preparation before the day of the experiment, followed by anthropometric and resting energy expenditure (REE) measurements in the morning of the experiment and a standardized breakfast. The anthropometric measurements included measurements of height using the stadiometer Seca 213 (Seca, Hamburg Germany) and weight using the Seca mBCA 515 (Seca, Hamburg, Germany); REE was assessed via inverse calorimetry with MetaLyser 3B (Cortex Biophysik GmbH, Leipzig, Germany) and MetaSoft Studio version 5.10 for computation of the REE. The MRI experiments were conducted on an Ingenia Elition X (Philips Healthcare, Best, The Netherlands) with software release 5.6.1 and were conducted in the early afternoon at least five hours after the breakfast. In the meantime, volunteers were instructed to not consume any food and drinks except water. The MRI experiment consisted of four baseline measurements before and 14 dynamic measurements after a break. During the break, the volunteers left the scanner room and continued fasting for 15min during the fasting control visit (C) or consumed an iso‐caloric meal during the meal intervention experiment. This separation into fasting control and meal intervention visits similar to previous cold intervention studies [22, 33] allowed to attribute observed effects specifically to the meal intervention. The meal intervention was defined as either protein‐rich (P) or non‐protein‐rich (nP), with carbohydrates and fat as the predominant macronutrients in the nP condition. The choice of intervention was based on each volunteer's strongest response in a previous study (study registry number: DRKS00026236). The energy content of the test meal was computed based on the individually assessed REE according to

(1) $$E_{\text{meal}}=1.2\times \frac{\text{REE}}{3}.$$

This accounted for a third of the volunteer's total daily energy expenditure multiplied by a physical activity level of 1.2 to reflect recumbent activity during the experiment. A visualization of the study design is presented in Figure 1.

**FIGURE 1:**
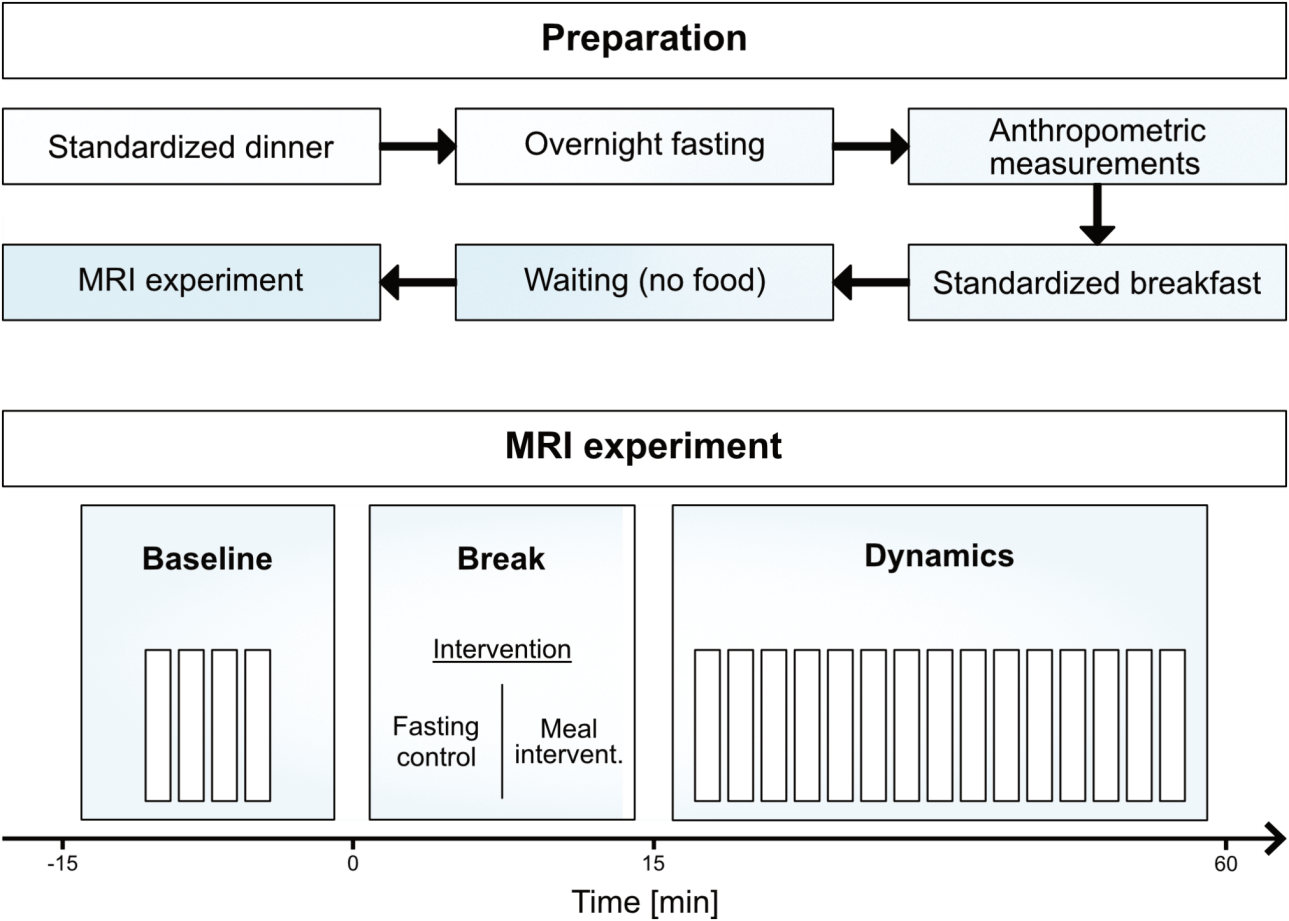
**Study design** The preparation starting on the day before the experiment included a standardized dinner and subsequent overnight fasting at least 12h before the volunteers received anthropometric and REE measurements and consumed a standardized breakfast. After that, volunteers were instructed to abstain from food until the start of the MRI experiment. The experiment consisted of four baseline measurements followed by a break and 14 dynamic measurements, represented by the vertical bars in the diagram. Each volunteer performed two experiments, in which they continued fasting (fasting control) or consumed an iso‐caloric meal (meal intervention). Abbreviation: REE, resting energy expenditure.

### 2.2 Volunteers

Ten healthy volunteers (five female, five male) who participated in the earlier study were enrolled for the MRI study. All volunteers gave informed written consent to participate in the study. In the meal intervention experiment, four volunteers consumed a P meal and six a nP meal. To compare the meal type, which created the strongest activation in the previous study to the other meal types, the volunteers were split into those who consumed a P meal (P group) and the others (nP group).

### 2.3 Data Acquisition

The MRI experiment consisted of repeated multi‐echo gradient echo measurements to enable water‐fat separation. The sequence employed six echoes and a bipolar radial SoS readout with pseudo‐golden angle spoke ordering [40] in the $k_{xy}$‐plane and 273 spokes per partition. All spokes per angle increment were acquired for all partitions before switching the angle in k‐space. Important scan parameters include the first echo time $T_{E,1}=1.27\;\text{ms}$, echo spacing $\Delta T_{E}=0.9\;\text{ms}$, number of echoes $nT_{E}=6$, repetition time $T_{R}=6.7\;\text{ms}$, flip angle α=3° and acceleration R=2 in feet‐head direction with SENSE. The small flip angle was employed to minimize $T_{1}$ bias effects [41, 42]. The scan duration per baseline and dynamic scan was $t_{i}=210\;\min\;\;s$. Pre‐scans to acquire a coil sensitivity map were performed prior to each baseline and dynamic repetition.

### 2.4 Data Processing

All MRI data was processed offline from raw k‐space according to the pipeline in Figure 2. A radial spoke alignment method was employed to address k‐space shifts for opposite spokes [43, 44]. Reconstruction was performed employing total variation (TV) regularization solving

(2) $$x=\underset{x^{\prime }}{\text{arg min}}{\left|\left|NSx^{\prime }-y\right|\right|}_{2}^{2}+\lambda {\left|\left|\text{TV}(x^{\prime })\right|\right|}_{1}$$

iteratively using the alternating direction method of multipliers (ADMM). In Equation 2, x is the complex‐valued multi‐echo image data, y is the k‐space data, N is the non‐uniform fast Fourier transform (NUFFT) operator, S is the coil sensitivity operator, and λ=0.5 is the regularization parameter. The coil sensitivity maps and a noise covariance matrix were computed from the pre‐scan using MRecon 4.4.8 (Gyrotools, Zurich, Switzerland). Reconstruction was performed using a public repository published earlier [45] with MATLAB R2022b (The Mathworks, Natick, Massachusetts, USA) and Julia 1.9.3 [46].

**FIGURE 2:**
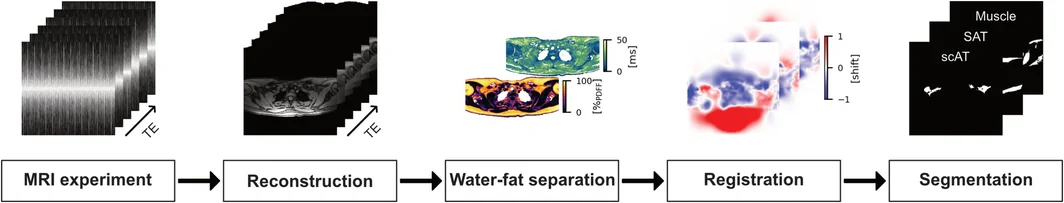
**Processing pipeline** Raw k‐space data was reconstructed offline using an iterative reconstruction. The resulting complex multi‐echo images were separated into water and fat images using a graph‐cut algorithm and quantitative PDFF and ${{\text{T}}_{2}}^{*}$ maps were computed. All baseline and dynamic scans were registered to the first dynamic using the water image and PDFF map. Water and fat images together with the quantitative PDFF and ${{\text{T}}_{2}}^{*}$ maps were used to create masks of scAT, SAT, and muscle tissue, which were subsequently post‐processed using joint thresholding and binary operations. Abbreviations: PDFF, proton density fat fraction; SAT, subcutaneous adipose tissue; scAT, supraclavicular adipose tissue.

Water and fat images were computed using WFS with a multi‐resolution graph‐cut algorithm on the complex multi‐echo data [47]. The employed water‐fat signal model at each voxel

(3) $$s(T_{\text{E}})=\left(\rho_{\text{W}}{\text{e}}^{-{wR_{2}}^{*}T_{\text{E}}}+c(T_{\text{E}})\rho_{\text{F}}{\text{e}}^{-{fR_{2}}^{*}T_{\text{E}}}\right){\text{e}}^{\text{i}2\pi f_{\text{B}}T_{\text{E}}}$$

with the proton densities $\rho_{\text{W}}$ of water and $\rho_{\text{F}}$ of fat, transverse relaxation times ${wT_{2}}^{*}={\left({wR_{2}}^{*}\right)}^{-1}$ of water and ${fT_{2}}^{*}={\left({fR_{2}}^{*}\right)}^{-1}$ of fat, and the field offset $f_{\text{B}}$ accounted for the six‐peak fat spectrum [48]

(4) $$c(T_{\text{E}})=\overset{6}{\underset{p=1}{\sum }}\alpha_{p}{\text{e}}^{\text{i}2\pi f_{p}T_{\text{E}}}$$

with the relative peak amplitude $\alpha_{p}$ and frequency offset $f_{p}$ of fat‐peak p. An established single‐${{\text{T}}_{2}}^{*}$ model with ${T_{2}}^{*}={wT_{2}}^{*}={fT_{2}}^{*}$ was employed to estimate a global ${{\text{T}}_{2}}^{*}$ map [49]. Before ${{\text{T}}_{2}}^{*}$ mapping, the complex‐valued multi‐echo data was spatially filtered with a 3D median filter of Size 2. The resulting water and fat images together with the PDFF and ${{\text{T}}_{2}}^{*}$ maps were used to create a mask of scAT using the nnU‐Net [50]. For that purpose, the network was trained based on manual segmentations available from ground truth labels used for for a previous study [39]. Here, scAT denotes the automatically segmented adipose tissue depot centered in the supraclacvicular region rather than a strictly anatomically confined supraclaviculuar department. The water image was further used to create masks of SAT and muscle tissue using the VIBESegmentator [51]. An example of the segmentations can be seen in Figure S1.

The water image and PDFF map were used to register [52, 53] each baseline and dynamic scan to the first dynamic after the break, which was defined to be the fixed image. The transformation field was then applied to the PDFF and ${{\text{T}}_{2}}^{*}$ maps to create co‐registered maps for each time point. This is crucial in order to apply the same masks to each time point and for analysis of changes on a per‐voxel basis. Final masks were created by joint thresholding [54] on the PDFF maps of all baseline and dynamic scans. The thresholds were $\text{PDFF}>35{\%}_{\mathrm{PDFF}}$ for the scAT mask, $\text{PDFF}>50{\%}_{\mathrm{PDFF}}$ for the SAT mask, and $\text{PDFF}<10{\%}_{\mathrm{PDFF}}$ for the muscle mask. Subsequently, all masks were processed with binary operations of filling holes, opening and erosion in that order. All processing was performed using Python 3.11.4 (Python Software Foundation, Wilmington, Delaware, USA) and the packages used are listed in Table S1.

### 2.5 Data Analysis

For each individual experiment, the time point $t_{0}=0\;\min$ was defined to be the start of the break, which means that the baseline scans are defined to have negative times and the dynamic scans positive times. PDFF and ${{\text{T}}_{2}}^{*}$ averages within each ROI were computed using the arithmetic mean and a single baseline value $q_{0},\;q$ being either PDFF or ${{\text{T}}_{2}}^{*}$, was computed as the median of all baseline scans. Subsequently, $q_{0}$ was subtracted from each dynamic scan $q_{i}$ as

(5) $${\tilde{q}}_{i}=q_{i}-q_{0}$$

to center the values around the baseline value.

To analyze the changes on a group level, all measurements were sorted into time bins of size 5min and averaged within each bin. For the analysis of the whole cohort, only bins with at least five data points were considered. Similarly, for the analysis of individual intervention types, only bins with at least three data points were considered. Histograms were computed with a bin width of $0.5{\%}_{\mathrm{PDFF}}$ and 1.0ms for PDFF and ${{\text{T}}_{2}}^{*}$, respectively. Difference histograms were computed on the difference between a dynamic scan and the average baseline. All histograms were normalized to analyze average histograms across the volunteers independent of ROI size.

To capture dynamic effects, an area under the curve (AUC) was computed of the dynamic values, centered around the average baseline. To take into account that initial increases of both PDFF and ${{\text{T}}_{2}}^{*}$ of unknown origin were observable after the break in many experiments independent of the intervention, the AUC was computed only from the 40min‐mark and thus named AUC‐40. For a voxel‐wise comparison, the AUC‐40 per voxel was computed and referenced to the baseline value of this voxel.

#### 2.5.1 Statistical Testing

All statistical analyses were performed assuming a significance level of p<0.05 as statistically significant.

Similarity between baseline histograms from control and activation was quantified using the pairwise Wasserstein distance on the histograms with equal bin width. The Wasserstein distance was computed as

(6) $$W=\underset{k}{\sum }\left|P_{k}-Q_{k}\right|,$$

with $P_{k}$ and $Q_{k}$ the cumulative sums of the normalized bin frequencies $p_{k}$ and $q_{k}$. This distance accounts for both shifts and differences of shapes [55]. To define an equivalence threshold, all pairwise Wasserstein distances $W_{ij}$ within an experiment type were computed, and the threshold δ was defined as the 95th percentile of these distances. Equivalence between control and activation baseline histograms was assessed using a sign‐flipping, permutation‐based one‐sided test for paired data. For each paired Wasserstein distance $W_{ij}$, the deviation from δ was calculated as $\Delta_{ij}=W_{ij}-\delta$. In each of 1000 permutations, the sign of these deviations were randomly flipped, and the mean was calculated. The p‐value was defined as the proportion of permuted mean deviations less than or equal the observed mean deviation.

For histogram differences, the mean value of each difference histogram was computed and assigned to the corresponding time point t. Differences between control and activation were evaluated using a two‐sided paired t‐test.

To assess the precision with which PDFF and ${{\text{T}}_{2}}^{*}$ changes can be detected independent of any systematic bias, the standard deviation (SD) and coefficient of variation (CV) of the mean PDFF and ${{\text{T}}_{2}}^{*}$ values were computed across the four baseline scans within each visit for scAT. The SD and CV were then averaged across all 20 sessions.

## 3 Results

### 3.1 Volunteer Characteristics

The median time between the fasting control and the meal intervention visits was 7.5d across all volunteers. During the meal intervention visit, four volunteers consumed a protein‐rich meal and six a non‐protein‐rich meal. The volunteer characteristics including age, BMI and REE, measured on the days of both visits, are listed in Table 1. Their mean BMI was $(\;23.6\pm 2.1)\;\mathrm{kg}\;m^{-2}$ and changed by $(\;0.0\pm 0.2)\;\mathrm{kg}\;m^{-2}$ over all volunteers between the fasting control and meal intervention visits. The change was $(\;0.1\pm 0.3)\;\mathrm{kg}\;m^{-2}$ in the P subgroup and $(\;-0.1\pm 0.1)\;\mathrm{kg}\;m^{-2}$ in the nP subgroup. The REE changed by $(\;15\pm 242)\;\mathrm{kcal}\;d^{-1}$ in all volunteers, by $(\;-6\pm 284)\;\mathrm{kcal}\;d^{-1}$ in the P group and by $(\;74\pm 199)\;\mathrm{kcal}\;d^{-1}$ in the nP group between visits.

**TABLE 1:** Volunteer characteristics five female and five male volunteers conducted two visits including a fasting control and an activation experiment including a meal intervention. Four volunteers received a protein‐rich meal.

Group	Age [y]	Intervention type	n	BMI [kg m 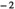 ]	REE [kcal d 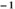 ]
All	25.1±2.8	Fasting control	10×C	23.6±2.1	1917±517
		Meal intervention	4×P,6×nP	23.6±2.1	1932±447
P	25.0±3.4	Fasting control	4×C	23.6±0.6	1771±422
		Meal intervention	4×P	23.7±0.8	1765±167
nP	25.2±2.4	Fasting control	6×C	23.6±2.6	1970±563
		Meal intervention	6×nP	23.5±2.7	2044±532

Abbreviations: BMI, body mass index; C, fasting control; nP, non‐protein‐rich food; P, protein‐rich food; REE, resting energy expenditure.

### 3.2 Stability of Measurements

The mean intra‐visit SD repeatability of mean PDFF in scAT was $0.24{\%}_{\mathrm{PDFF}}$ with a CV of 0.3%. For ${{\text{T}}_{2}}^{*}$, repeatability was 0.91ms with a CV of 3.3% in scAT. The distributions of SD and CV in scAT are visualized in Figure S2.

### 3.3 Example Dynamic Mapping Results

The coronal slices in the top row of Figure 3b depict the baseline PDFF of one volunteer for the fasting control and meal interverntion experiments. Below, the first echo magnitudes of the respective baseline are superimposed by the scAT PDFF difference at 40 and 60min. There were areas of slight changes in the fasting control experiments at both time points and in the meal intervention experiment at 40min. At 60min, some areas of strong PDFF decrease are visible after meal intervention. The respective histograms in Figure 3a show that the baseline PDFF was similar in the fasting control and meal activation experiments. In the fasting control experiment, the differences were centered around $\Delta \text{PDFF}=0{\%}_{\mathrm{PDFF}}$ while the difference histograms show a shift towards decreasing PDFF in the meal intervention experiment. Average PDFF over time is plotted in Figure 3c and shows an initial increase of scAT PDFF in the fasting control experiment after the break with subsequent decrease close to the baseline value. In contrast, PDFF decreased below the baseline value in the meal intervention experiment and stayed decreased until the end of the experiment. On a per‐voxel basis, the dynamics were analyzed using the ${\text{PDFF}}_{\mathrm{AUC}-\mathrm{40}}$ in Figure 3d. In the fasting control experiment, most voxels had a ${\text{PDFF}}_{\mathrm{AUC}-\mathrm{40}}$ close to 0 while most voxels had a negative ${\text{PDFF}}_{\mathrm{AUC}-\mathrm{40}}$ in the meal intervention experiment. Here, a trend for voxels with higher baseline PDFF to have a lower ${\text{PDFF}}_{\mathrm{AUC}-\mathrm{40}}$ is observed.

**FIGURE 3:**
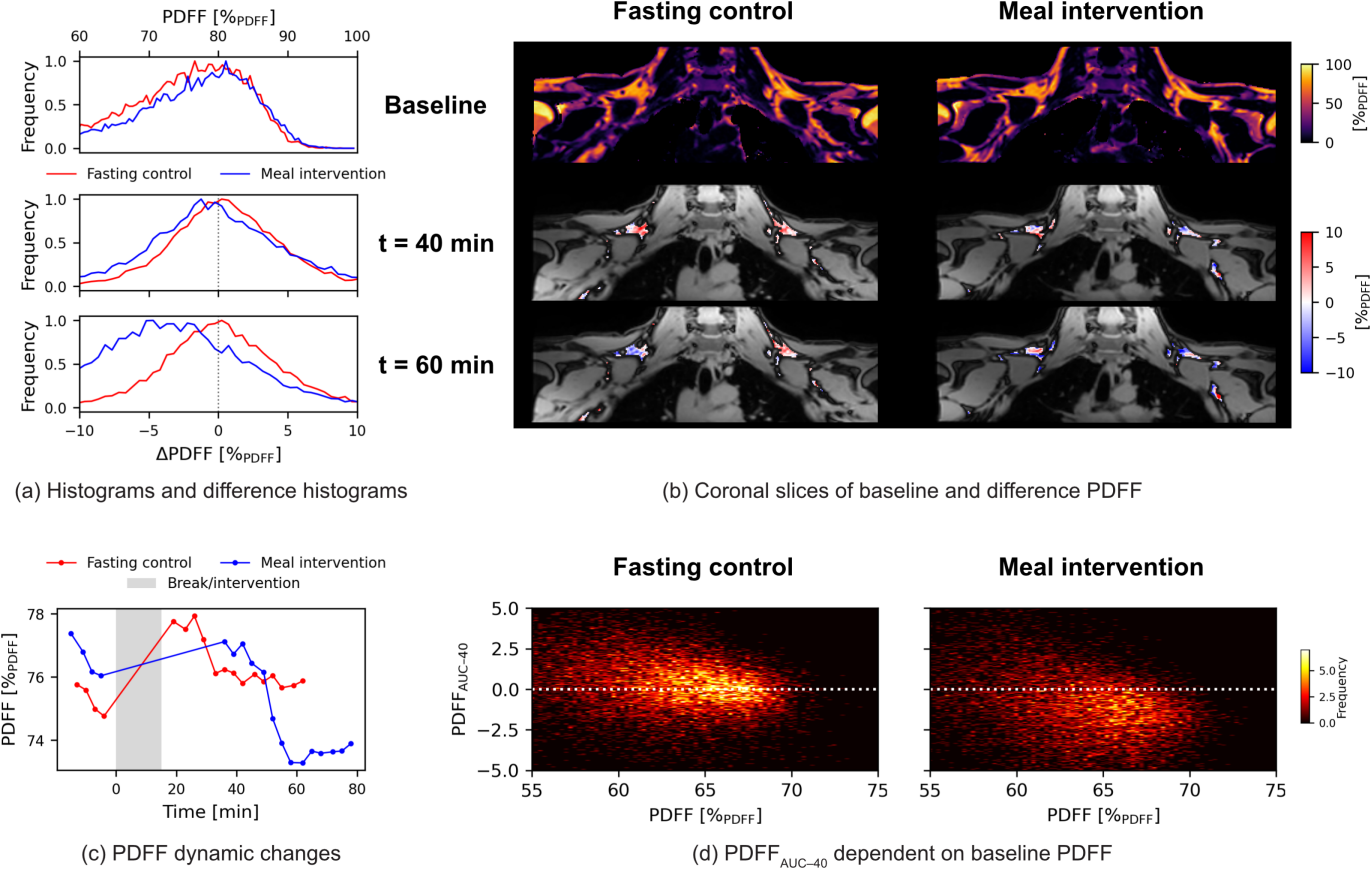
**Example dynamic mapping results: PDFF mapping** (a) The top histogram displays the distribution of scAT PDFF for one volunteer at baseline for the fasting control experiment in red and meal intervention experiment in blue. Below are difference histograms to the baseline for two time points. (b) The top row contains coronal slices of the PDFF map of one volunteer for the fasting control experiment on the left and the meal intervention experiment on the right. Difference maps within scAT are superimposed over the first echo magnitude image below for the two time points corresponding to the histograms in (a). Blue voxels indicate a decrease of PDFF compared to baseline, red voxels indicate increasing PDFF. (c) The change of mean PDFF of one volunteer is plotted for the fasting control experiment in red and the meal intervention experiment in blue. The baseline scans are defined at negative times and the break or intervention started at 0 min and is visualized by the gray background. (d) The plots visualize the dynamic PDFF change of one volunteer using the parameter ${\text{PDFF}}_{\mathrm{AUC}-\mathrm{40}}$ per voxel compared to its baseline value for fasting control on the left and meal activation on the right. No change is indicated by the dotted white line, values below the line indicate a decrease of PDFF. The intensity of the plot represents the number of voxels within scAT with a particular baseline PDFF and ${\text{PDFF}}_{\mathrm{AUC}-\mathrm{40}}$. Abbreviations: PDFF, proton density fat fraction; scAT, supraclavicular adipose tissue.

The baseline coronal slices of ${{\text{T}}_{2}}^{*}$ in Figure 4b had less heterogeneity in values compared to the slices of PDFF in Figure 3b. However, the differences at 40 and 60min in scAT revealed more apparent differences between fasting control and meal intervention experiments compared to PDFF. While there was no clear pattern in the fasting control experiment, big areas of large ${{\text{T}}_{2}}^{*}$ decrease were visible in the meal intervention experiment at both time points. The difference histograms in Figure 4a support this finding with a small shift towards smaller values at 40 min but a large shift at 60min after meal intervention while the difference in the fasting control experiment is centered around Δ ${{\text{T}}_{2}}^{*}$ =0ms. The ${{\text{T}}_{2}}^{*}$ changes over time in Figure 4c showed a similar behavior to the PDFF dynamics with an initial increase in the fasting control experiment with subsequent decrease around the baseline value. ${{\text{T}}_{2}}^{*}$ in the meal intervention experiment showed a decrease compared to baseline starting at around 45min. The per‐voxel analysis in Figure 4d showed that most voxels did not show dynamic effects in the fasting control experiment while most voxels exhibited a negative ${{\text{T}}_{2}}^{*}$ 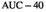 in the meal intervention experiment. Voxels with higher baseline ${{\text{T}}_{2}}^{*}$ tended to have a lower ${{\text{T}}_{2}}^{*}$ 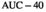.

**FIGURE 4:**
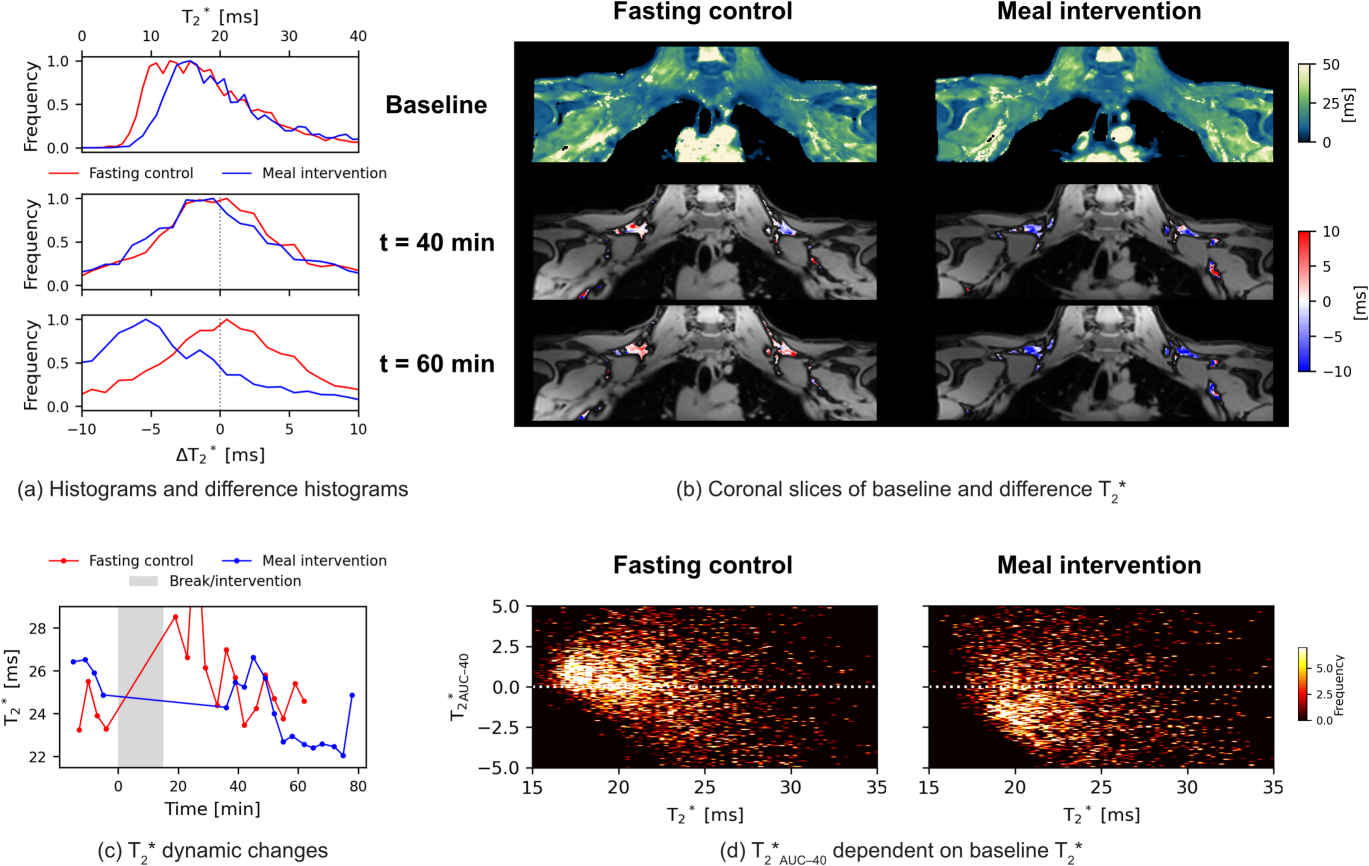
**Example dynamic mapping results:** ${{\text{T}}_{2}}^{*}$ **mapping** (a) The baseline histogram in the top displays the distribution of scAT ${{\text{T}}_{2}}^{*}$ in one volunteer for both the fasting control experiment in red and the meal intervention experiment in blue. The two difference histograms below show the difference of scAT ${{\text{T}}_{2}}^{*}$ at 40 and 60min compared to baseline with negative values indicating a decrease of ${{\text{T}}_{2}}^{*}$. (b) The coronal slices of baseline ${{\text{T}}_{2}}^{*}$ of one volunteer for the fasting control experiment on the left and meal intervention experiment on the right show the quality of ${{\text{T}}_{2}}^{*}$ mapping in the neck area. Below, difference maps of ${{\text{T}}_{2}}^{*}$ are superimposed on the respective first echo magnitude images, visualizing the change of ${{\text{T}}_{2}}^{*}$ in scAT compared to baseline per voxel. Red coloring means an increase, blue a decrease. (c) The dynamic changes of scAT ${{\text{T}}_{2}}^{*}$ are plotted in red for the fasting control experiment and in blue for the meal intervention experiment of one volunteer. The baseline values to the left of the gray area, which indicates the time of break or intervention, are at negative times. (d) The changes of scAT ${{\text{T}}_{2}}^{*}$ per voxel in one volunteer quantified by ${{\text{T}}_{2}}^{*}$ 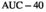 compared to the baseline ${{\text{T}}_{2}}^{*}$ is shown for the fasting control experiment on the left and the meal intervention experiment on the right. The pixel intensity represents the number of voxels of the scAT mask with the specific combination of baseline and dynamic ${{\text{T}}_{2}}^{*}$. Abrreviation: scAT, supraclavicular adipose tissue.

### 3.4 Group Dynamic Mapping Results

The averages of PDFF (first row) and ${{\text{T}}_{2}}^{*}$ (second row) over time sorted into bins of 5min over the whole cohort are plotted in Figure 5a. The averages are plotted for the scAT (left column), SAT (center column) and muscle (right column) ROIs. scAT PDFF is increasing in the fasting control experiment after the break before decreasing below baseline at 40min and staying decreased at $(\;-0.3\pm 0.2){\%}_{\mathrm{PDFF}}$ to 60min. In the meal intervention experiment, PDFF decreased without increase after the intervention to values lower than in the fasting control experiment with the lowest at $-1.5{\%}_{\mathrm{PDFF}}$ at 60min. A similar behavior is observable in the SAT ROI, with fasting control PDFF from 40 to 60min at $(\;-0.2\pm 0.1){\%}_{\mathrm{PDFF}}$ and a minimum PDFF in the meal intervention experiment of $-0.9{\%}_{\mathrm{PDFF}}$ at 60min. In the muscle ROI, PDFF remained stable over time. Similarly to PDFF in scAT, scAT ${{\text{T}}_{2}}^{*}$ increased to a maximum of 1.3ms before decreasing to below baseline at 40min and staying decreased at (−0.2±0.5)ms until 60min in the fasting control experiment. The ${{\text{T}}_{2}}^{*}$ in the meal intervention experiment decreased immediately after the intervention, remained decreased and decreased further than in the fasting control experiment to a maximum decrease of −3.2ms at 65min. In both the SAT and muscle ROIs, ${{\text{T}}_{2}}^{*}$ did not exhibit substantial decreases below baseline. Those findings are supported by the quantification of late dynamics in Figure 5b using the AUC‐40. For both PDFF and ${{\text{T}}_{2}}^{*}$, AUC‐40 is only markedly negative in scAT after the meal intervention. The AUC‐40 in scAT after meal intervention is significantly different to SAT and muscle for both PDFF and ${{\text{T}}_{2}}^{*}$, ${{\text{T}}_{2}}^{*}$ 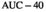 also is significantly different to scAT in the fasting control experiment.

**FIGURE 5:**
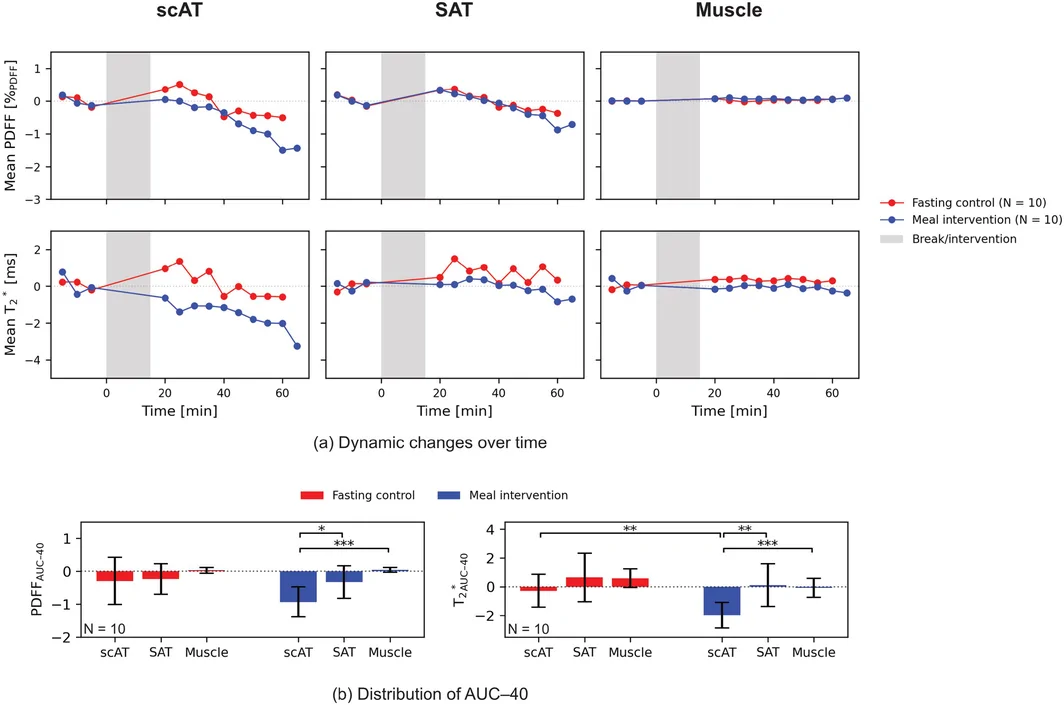
**Group dynamic mapping results** (a) The average PDFF (top row) and ${{\text{T}}_{2}}^{*}$ (bottom row) over the whole cohort is plotted, from left to right, for the scAT, SAT, and muscle ROIs. The red line represents the dynamics from the fasting control experiments and the blue line the meal intervention experiments. The gray area marks the time of break or intervention. (b) The average ${\text{PDFF}}_{\mathrm{AUC}-\mathrm{40}}$ and its standard deviation are plotted in the left diagram for scAT, SAT, and muscle from left to right in red for the fasting control experiments and in blue for the meal intervention experiments. The average ${{\text{T}}_{2}}^{*}$ 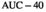 and its standard deviation are plotted in the right diagram for scAT, SAT and muscle from left to right in red for the fasting control experiments and in blue for the meal intervention experiments. Significant differences between groups are indicated with asterisks. *p<0.05, **p<0.01, ***p<0.001. Abbreviations: AUC, area under the curve; PDFF, proton density fat fraction; ROI, region of interest; SAT, subcutaneous adipose tissue; scAT, supraclavicular adipose tissue.

The PDFF baseline histograms (top row) and difference histograms of the whole cohort at 40min (center row) and at 60min (bottom row) are displayed in Figure 6a. According to the Wasserstein distance, the baseline histograms were statistically significantly similar at baseline with p<0.001 for PDFF in all three ROIs. After 40min, scAT PDFF was decreased slightly in both fasting control and meal intervention experiments with no significant difference and bigger decrease after 60min with a significant difference between fasting control and meal intervention experiments of p=0.003. PDFF in SAT showed smaller decreases while in muscle PDFF was stable and there were no significant differences between fasting control and meal intervention experiments. The same layout in Figure 6b for ${{\text{T}}_{2}}^{*}$ displays similar baseline distributions between fasting control and meal intervention experiments with similar histograms at baseline with p<0.01 for ${{\text{T}}_{2}}^{*}$ in all ROIs. Here, the difference histograms reveal that ${{\text{T}}_{2}}^{*}$ decreased mostly in the late dynamic of the meal intervention experiment in scAT with a significant difference between fasting control and meal intervention of p<0.001 while other differences remain close to 0.

**FIGURE 6:**
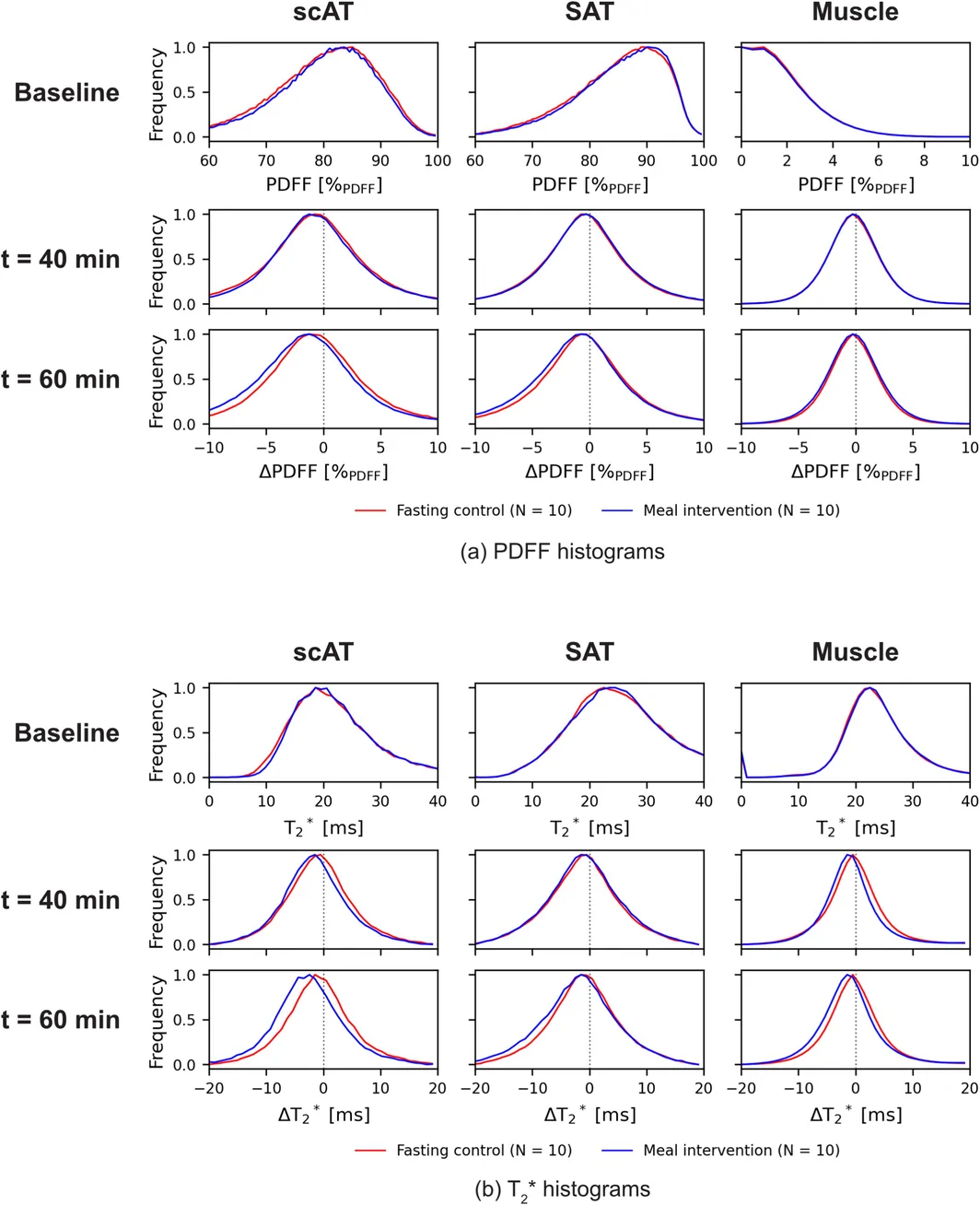
**Average histogram results** (a) The plots in the top row show the baseline distribution of PDFF on the cohort level in scAT on the left, SAT in the center and muscle on the right for the fasting control experiment in red and the meal intervention experiment in blue. It is worth noting that the range on the x‐axis is different for the muscle ROI compared to the other plots. The difference histograms below at 40 and 60min visualize the PDFF difference to baseline in the respective ROIs at different time points for the whole cohort. (b) The plots show the baseline distribution and differences of ${{\text{T}}_{2}}^{*}$ using the same arrangement as in (a). Notably, the *x*‐axis of baseline plots is the same for all three ROIs. Abbreviations: PDFF, proton density fat fraction; ROI, region of interest; SAT, subcutaneous adipose tisue; scAT, supraclavicular adipose tissue.

The voxel‐wise dynamic ${\text{PDFF}}_{\mathrm{AUC}-\mathrm{40}}$ are displayed in Figure 7a for the scAT and SAT ROIs. The average PDFF dynamic change is −0.43 in the fasting control experiment for scAT and −0.21 in SAT. After meal intervention, there is an average change of −0.93 in scAT and −0.44 in SAT. The value of baseline PDFF did not have an influence on the observed ${\text{PDFF}}_{\mathrm{AUC}-\mathrm{40}}$ per voxel. Similarly, for ${{\text{T}}_{2}}^{*}$ in Figure 7b the changes are in the fasting control experiment −0.11 for scAT and 0.06 for SAT and after meal intervention −1.00 for scAT and −0.15 for SAT. Notably, ${{\text{T}}_{2}}^{*}$ showed a stronger dynamic decrease for voxels with higher baseline ${{\text{T}}_{2}}^{*}$. No trends for changes were observable in muscle as illustrated in Figure S3.

**FIGURE 7:**
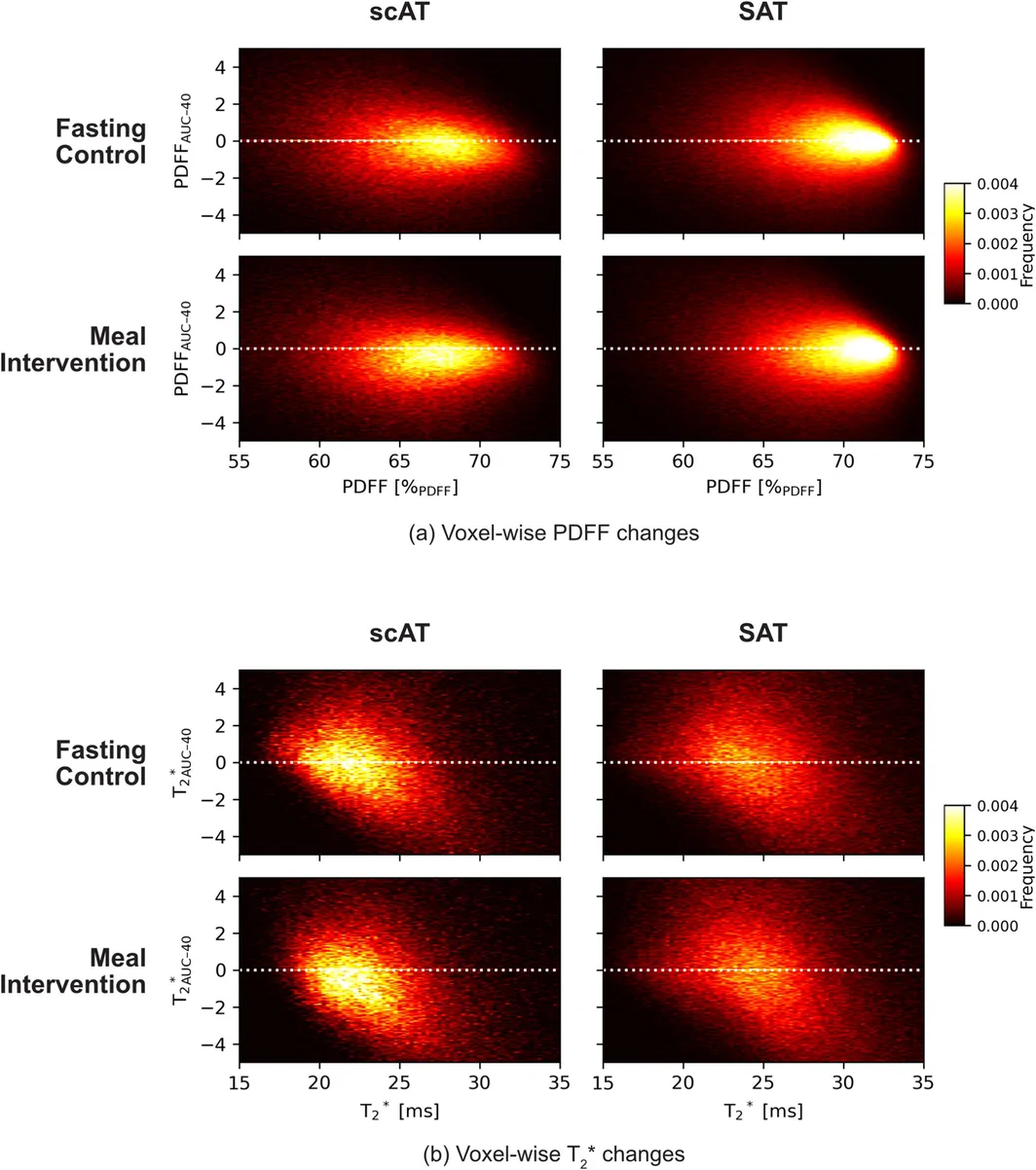
**Voxel‐wise change results** (a) Each plot shows the distribution of voxels according to their baseline PDFF and ${\text{PDFF}}_{\mathrm{AUC}-\mathrm{40}}$ over the whole cohort. The distributions are shown for scAT on the left and SAT on the right for both experiments, fasting control in the top row and meal intervention in the bottom row. (b) The distributions of baseline ${{\text{T}}_{2}}^{*}$ and ${{\text{T}}_{2}}^{*}$ 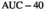 are displayed using the same layout as in (a). Abbreviations: PDFF, proton density fat fraction; SAT, subcutaneous adipose tissue; scAT, supraclavicular adipose tissue.

### 3.5 Intervention Comparison

In an exploratory subgroup analysis, PDFF and ${{\text{T}}_{2}}^{*}$ dynamics were compared between volunteers from the P and nP groups. Given the small subgroup sizes of four and six volunteers, respectively, this comparison is not statistically powered and is reported using the subgroup averages over time in Figure S4, the distributions of AUC per subgroup in Figure S5, and histogram differences in Figure S6. Both PDFF and ${{\text{T}}_{2}}^{*}$ exhibit steeper decreases in volunteers of the P group compared to nP while the control experiments did not reveal obvious differences between both groups. However, this analysis should be interpreted as hypothesis‐generating only.

## 4 Discussion

The present study reports meal‐induced PDFF and ${{\text{T}}_{2}}^{*}$ decrease in human scAT. The study employs a methodological framework combining a motion‐robust acquisition and reconstruction methodology with advanced image analysis tools to track small changes in PDFF and ${{\text{T}}_{2}}^{*}$ in the human scAT. The methodology is applied to a study, where the same subjects underwent measurements both under fasting control and after meal intervention conditions.

Dual‐echo or multi‐echo acquisitions for WFS have been the most common choice to study the lipid composition of scAT using MRI. A useful parameter in that aspect is the signal‐weighted fat fraction from dual‐echo Dixon and the PDFF from multi‐echo Dixon acquisitions. In the present work, a six‐echo gradient echo acquisition was employed to quantify the fat content, and a lower PDFF in scAT compared to SAT was found in line with many previous results [13, 17, 18, 24, 25]. The WAT‐dominated SAT contains large lipid droplets which results in a higher fat content compared to BAT‐containing scAT, which consists of smaller lipid droplets with a higher mitochondrial count and more excessive vascularization [56]. This explains the lower observed PDFF in scAT, though a previous analysis reported a substantially different distribution of PDFF with a high number of voxels inside the scAT ROI with $<50{\%}_{\mathrm{PDFF}}$ using a histogram than reported in this work [57]. However, the direct comparison of quantitative values between studies is rendered difficult by different means of ROI delineation. The study in question employed a semi‐manual segmentation while the ROI segmentation was completely automated in the present study. Also the decreased ${{\text{T}}_{2}}^{*}$ at baseline in scAT compared to SAT is explained by the morphological differences, since scAT, with its vascularization, small lipid droplets and iron‐rich mitochondria, is more heterogeneous and more paramagnetic than SAT, and therefore has higher magnetic susceptibility, which decreases ${{\text{T}}_{2}}^{*}$ [15, 23]. Previous studies reported smaller ${{\text{T}}_{2}}^{*}$ in scAT compared to SAT in line with the present results [13, 25].

Upon cold activation, PDFF has widely been reported to decrease in scAT during cooling [17, 31, 32, 33] and after the cooling period [25, 26, 28, 29, 30, 57, 58, 59] with only few studies finding no significant PDFF decrease after cooling [13, 22, 27, 28]. The present work is the first study to systematically analyze the effects of a meal‐induced activation on the WFS‐MRI‐derived parameters of scAT in humans. Even though the underlying activation pathways differ with cold exposure primarily triggering sympathetic nervous system activity and norepinephrine‐mediated thermogenesis [60], whereas meal intake activates adipose tissue through postprandial hormonal signaling via secretin [7, 61], ultimately promoting lipid mobilization and increased mitochondrial activity within adipocytes [62, 63]. These shared effects could explain why a similar decrease of scAT PDFF was observable, reflecting a reduction in intracellular lipid content. Notably, PDFF of both scAT and SAT were decreasing upon meal‐induced activation in the present study, an effect observed before in some cold activation experiments [31, 32, 33] while others did not find significant changes in SAT [13, 17, 30, 57, 58, 64]. It is worth noting that PDFF in both adipose tissues did also decrease in the control experiments. This is consistent with previous studies in which a warm control experiment was performed additionally to the cold activation [13, 33]. In light of the observed decrease of PDFF in both adipose tissues under fasting control as well as meal intervention conditions, it is important to note that volunteers were instructed to abstain from food over several hours in the control experiments in the present study as well as in previous studies. This intermittent fasting can induce lipolysis in WAT [65], which effectively reduces PDFF in WAT‐containing tissues. This could explain the PDFF decrease in SAT, which follows the same trend and magnitude for most of the measured time in both the fasting control and the meal intervention experiments. Similarly, scAT PDFF decreased by a similar magnitude in the fasting control experiment compared to the decrease in SAT but to a greater extent after the meal intervention. The additional PDFF decrease likely reflects meal‐induced BAT activation in scAT. Thus, the effect of meal‐induced activation in the present cohort accounts for a decrease of $∼1{\%}_{\mathrm{PDFF}}$ in scAT at 60min after the activation. Importantly, PDFF in the muscle ROI as a reference tissue, where no reaction to the meal activation is expected, remained stable over the course of the experiment, similar to previous studies [13, 28, 31, 33].

${{\text{T}}_{2}}^{*}$ has not been reported on during cold activation experiments as widely as the fat fraction. Several studies only employed dual‐echo gradient echo acquisitions which cannot provide a reliable estimate of the ${{\text{T}}_{2}}^{*}$ map. But also some studies which employed multi‐echo acquisitions did not report on ${{\text{T}}_{2}}^{*}$ results. Since ${{\text{T}}_{2}}^{*}$ measurements are known to have low reproducibility, especially in a complex anatomy such as the neck [36, 37], some studies might have opted not to include ${{\text{T}}_{2}}^{*}$ analysis. The present study, however, is the first to combine a radial SoS acquisition with robust $B_{0}$ field mapping using a graph‐cut algorithm [47] for reliable ${{\text{T}}_{2}}^{*}$ estimation in the neck area to track ${{\text{T}}_{2}}^{*}$ changes in scAT over time [37]. Upon cold activation, two studies found changes in scAT using the blood oxygenation level dependent (BOLD) effect [23, 24] which relies on ${{\text{T}}_{2}}^{*}$‐weighted imaging and expresses changes in oxygenation levels. Using ${{\text{T}}_{2}}^{*}$ quantification techniques, the results have been mixed with some studies reporting on decreasing scAT ${{\text{T}}_{2}}^{*}$ [32, 58] but more studies report on no significant effects [13, 22, 25, 26, 57]. The results in the present study conversely indicate a ${{\text{T}}_{2}}^{*}$ decrease in scAT upon meal‐induced activation with no substantial changes in the fasting control experiment or in SAT. In fact, the ${{\text{T}}_{2}}^{*}$ decrease in scAT was more dominant than the PDFF decrease, and ${{\text{T}}_{2}}^{*}$ changes were significantly larger after meal intervention compared to ${{\text{T}}_{2}}^{*}$ changes after the fasting control experiment, suggesting that ${{\text{T}}_{2}}^{*}$ serves as a more sensitive biomarker for BAT activation than PDFF, when compromising effects are handled properly. Next to the aforementioned issues during acquisition and data processing regarding ${{\text{T}}_{2}}^{*}$ quantification which were addressed in the present study and the different mechanism of BAT activation, also ROI selection may play a role influencing the results. While previous studies often relied on manual or semi‐manual segmentations or manually placed circular ROIs, which holds the risk of inducing biases into the measurements, the present study analyzed the whole scAT using fully automated processing. Since especially ${{\text{T}}_{2}}^{*}$ can be very heterogeneous within scAT, this might be an additional explanation to the mixed results of previous studies. The ${{\text{T}}_{2}}^{*}$ decrease can be explained by multiple processes happening in BAT during activation, including an increase in perfusion and resulting inflow of iron‐containing hemoglobin which shortens ${{\text{T}}_{2}}^{*}$, vessel dilation and lipolysis which increases local susceptibility [15, 21]. It is worth noting that considering the mechanisms mentioned, both SAT and muscle are considered to be reference tissues regarding ${{\text{T}}_{2}}^{*}$ changes. While this study does not report on changes in both tissues and most studies support this [13, 32, 57], one study found a decrease in SAT ${{\text{T}}_{2}}^{*}$ after cold‐induced activation [58].

A single ${{\text{T}}_{2}}^{*}$ decay for water and fat was used for the primary analysis, consistent with established techniques for field mapping and WFS [49, 66]. Estimating independent ${wT_{2}}^{*}$ and ${fT_{2}}^{*}$ values with a dual‐${{\text{T}}_{2}}^{*}$ model is known to be considerably less stable than a shared single‐${{\text{T}}_{2}}^{*}$ model, particularly with the limited echo sampling of six echoes and a maximum $T_{\text{E}}$ well below 20ms employed in this study, which is required to preserve the temporal resolution needed for dynamic imaging. Under the present $T_{\text{E}}$ settings, a dual‐${{\text{T}}_{2}}^{*}$ model, which introduces an additional free parameter per voxel, is poorly conditioned at typical signal‐to‐noise ratios achieved in vivo, and has been shown to increase noise and instability relative to a single‐${{\text{T}}_{2}}^{*}$ model in liver [67, 68]. Consistent with this, a previous study showed that even single‐${{\text{T}}_{2}}^{*}$ estimation in scAT becomes less precise when only six echoes are sampled [18]. This limitation was handled in the present study by carefully designing the employed acquisition, reconstruction, and processing according to a previously validated pipeline [37]. Extending the number of sampled echoes to stabilize dual‐${{\text{T}}_{2}}^{*}$ fitting would lengthen the scan, thereby reducing temporal resolution, and increase motion sensitivity, both undesirable for the dynamic protocol used here. Therefore, the single‐${{\text{T}}_{2}}^{*}$ model was considered to be more robust for the present acquisition. It is worth noting, that comparable cold‐activation studies, that investigated scAT using MRI, did not employ a jointly‐fitted dual‐${{\text{T}}_{2}}^{*}$ model when ${{\text{T}}_{2}}^{*}$ was reported on.

The precision of detecting PDFF and ${{\text{T}}_{2}}^{*}$ changes was assessed using the intra‐subject change under the actual in vivo conditions including motion, $B_{0}$ inhomogeneities and fluctuations, and physiological variability. The resulting repeatability SD for PDFF of $0.24{\%}_{\mathrm{PDFF}}$ is six times smaller than the observed PDFF decrease in the meal intervention experiment at 60min, and the repeatability SD for ${{\text{T}}_{2}}^{*}$ of 0.91ms is more than three times smaller than the observed decrease. This indicates that both effects clearly exceed the noise floor of the pipeline used throughout this study. This conclusion is supported by different additional, independent evidence included in the study design: first, the inclusion of a fasting control visit, which showed that PDFF and ${{\text{T}}_{2}}^{*}$ remained stable or decreased less in the absence of a meal intervention; second, the muscle ROI, which is not expected to respond to the intervention and shows stable values across all dynamic time points in both visits; and third, the consistent temporal decrease of PDFF and ${{\text{T}}_{2}}^{*}$ across the dynamic scans, which is inconsistent with the trends being artifacts of measurement noise. Furthermore, the reliable estimation of ${{\text{T}}_{2}}^{*}$ in the supraclavicular anatomical region with the temporal resolution acquired in the present study is nontrivial and not routinely attempted in comparable studies [17, 28, 31, 33, 58]. The ability to perform this analysis reflects the previously validated stability of the motion‐robust acquisition, reconstruction, and processing pipeline used in the present study [37].

In the fasting control experiment, PDFF and ${{\text{T}}_{2}}^{*}$ in both scAT and SAT initially increased after the break. Since the values in the muscle ROI did not show a similar behavior, this is likely related to physiological processes specific to adipose tissues rather than a global measurement artifact. Standing up and walking out of the scanner room after lying in the supine position on the scanner bed is expected to reduce effective perfusion in adipose tissues due to sympathetic vasoconstriction [69, 70], leading to a relative reduction of water signal and, consequently, apparently increased PDFF. Similarly, the reduction in intravascular blood volume may reduce local susceptibility heterogeneity, which could increase apparent ${{\text{T}}_{2}}^{*}$ [71]. In addition, potential mild cooling while leaving the scanner could trigger sympathetic vasoconstriction, reducing the blood flow in adipose tissues before BAT activation [70], again leading to the same PDFF and ${{\text{T}}_{2}}^{*}$ effects described before.

The scAT PDFF histograms reported in this study differed from some previously reported distributions [57]. At baseline and after activation, PDFF histograms in the present study show a similar shape compared to previous work in the higher PDFF range, but earlier work reported that many scAT voxels contained less than $50{\%}_{\mathrm{PDFF}}$ [57]. While PDFF quantification can be affected by differences in acquisition, reconstruction or the fat model, the shift of the apparent distribution towards lower PDFF is likely because the earlier study employed manual segmentation of the scAT ROI, which may have caused inclusion of surrounding lean tissue and vessels into the scAT ROI.

On a per‐voxel basis, the scAT PDFF dynamics in this study were independent of the initial PDFF of that voxel. In contrast, a stronger decrease for voxels with higher initial PDFF was found in a previous study when only the difference between the baseline and the PDFF after the meal intervention were analyzed [72]. Similar results were obtained in the difference between before and after cold exposure [57]. Likewise, ${{\text{T}}_{2}}^{*}$ decreased more strongly in voxels with initially higher ${{\text{T}}_{2}}^{*}$ after the meal challenge [72], a finding reproduced in the present study, albeit to a lesser extent. However, the dynamic changes reported in the present study measured a different parameter, the AUC starting 40min after the meal activation, reflecting persistent PDFF or ${{\text{T}}_{2}}^{*}$ decrease over time rather than a single pre‐post difference. Consequently, direct comparisons between both metrics should be interpreted with caution.

The exploratory comparison between meal types suggested a trend towards larger PDFF and ${{\text{T}}_{2}}^{*}$ decreases with the protein‐rich meal, consistent with known differences in diet‐induced thermogenesis between macronutrients [73]. However, with only four and six volunteers per group, this observation is not supported by sufficient statistical power and should be treated as hypothesis‐generating. Confirmation would require a dedicated, adequately powered study.

It is worth noting that there are several limitations to this study. First, the cohort size of ten volunteers is small in absolute terms. It is, however, comparable to cohort sizes commonly used in prior human BAT‐activation MRI studies, which have typically ranged from eight to 25 volunteers, with several cold‐activation studies using cohorts of similar size [13, 17, 30, 31, 32, 33, 57, 58]. The volunteers in the present study were recruited from a previously completed cohort, and further recruitment from this pool was not possible. Generalizability could nonetheless be further improved with independently recruited, larger cohorts in future work, particularly to resolve subgroup effects, like meal type, with adequate statistical power. Second, the choice of meal was based on the individual volunteer's response in the original, unpublished study. This might have induced a bias in assigning the protein‐rich meal to those volunteers who show a stronger postprandial effect. Third, since the volunteers had to leave the scanner for meal consumption, physiological effects of the motion could not be discarded and might have influenced the data of early dynamic scans. Fourth, no temporal $B_{0}$ correction methods were used in the reconstruction process. Compensating for temporal $B_{0}$ fluctuations on the reconstruction level could stabilize ${{\text{T}}_{2}}^{*}$ quantification further. Fifth, comparing to most previous studies investigating BAT activation using MRI, the ROI selection was fully automated and, thus, reproducible. While this marks an improvement compared to the previous studies, it reduced the control over the analyzed tissue and renders the comparison to previous studies difficult. Sixth, between‐visit differences in pre‐scan physiological conditions, including the exact interval between breakfast and MRI experiment, physical activity, outside temperature and stress, may have influenced the measured baseline state. However, the similarity of baseline PDFF and ${{\text{T}}_{2}}^{*}$ histograms between visits mitigates this concern by supporting comparable imaging‐derived starting states.

## 5 Conclusions

Using robust acquisition, data processing, and analysis, this study showed for the first time that consumption of a high‐caloric meal reduced PDFF and ${{\text{T}}_{2}}^{*}$ in human scAT, while no similar dynamics were observed in reference tissues. By employing a two visit study design including a fasting control experiment, PDFF and ${{\text{T}}_{2}}^{*}$ changes could be differentiated from fasting effects and attributed to the meal intervention. While PDFF remains an important parameter to observe scAT activation, this study showed that ${{\text{T}}_{2}}^{*}$ may serve as the more sensitive biomarker to track dynamic changes of scAT upon meal activation. Together, this study can help to understand the effects of diet on adipose tissues and can elicit future work investigating PDFF and ${{\text{T}}_{2}}^{*}$ changes upon meal interventions using different meal types in larger cohorts.

## Author Contributions

Conceptualization: M.W., M.K., T.S., D.C.K. Data Curation: J.R. Formal analysis: J.R., T.D. Funding acquisition: M.K., T.S., D.C.K. Investigation: J.R., T.D., D.J. Methodology: J.R., M.W., J.S., D.C.K. Project administration: M.K., D.J., D.C.K. Resources: D.C.K. Software: J.R., J.S. Supervision: M.K., T.S., D.C.K. Validation: J.R., T.D. Visualization: J.R. Writing – original draft preparation: J.R., T.D. Writing – review and editing: J.R., T.D., M.W., J.S., M.K., D.J., T.S., D.C.K.

## Funding

The present research was supported by the German Research Foundation (project numbers 455422993/FOR5298‐iMAGO‐P1 and 455422993/FOR5298‐iMAGO‐P5).

## Conflicts of Interest

The authors declare no conflicts of interest.

## Supporting information

iMAGO_dynamics_revision1_supplementary.pdf

tableS1.tex
