# Supplementary material for: Meal‐Induced Proton Density Fat Fraction and T2∗ Decrease in Supraclavicular Adipose Tissue
Source: NMR Biomed. 2026 Oct 5;39(11):e70414. doi: 10.1002/nbm.70414 (PMC13639107; doi:10.1002/nbm.70414)
Supplement: Supplementary file 1 [file NBM-39-e70414-s002.pdf]

Meal-induced proton density fat fraction and $T_2{}^*$ decrease in supraclavicular adipose tissue

Supplementary information

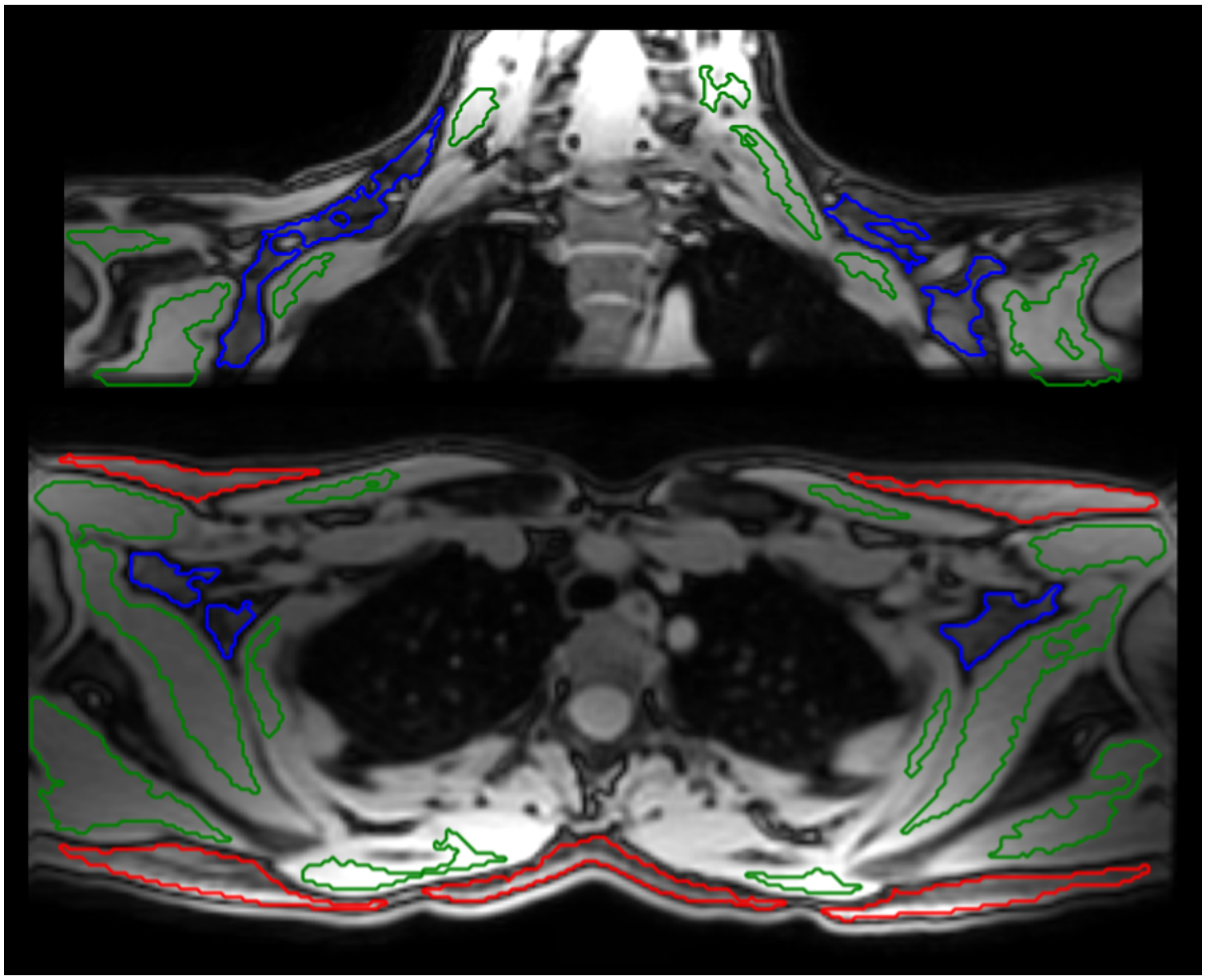

Figure S1: **Segmentation example**
The images represent example coronal (top) and axial (bottom) slices of the first echo magnitude of one volunteer. The highlighted areas are the automatic segmentations of the scAT ROI using the nnU-Net in blue and the SAT ROI in red and muscle ROI in green using the VIBESegmentator.

Table S1: **Used Python packages**
Below is a list of the Python packages used for data processing and analysis this study in alphabetical order. All packages are listed with their respective versions.

Package name	Package version
Joblib	1.3.2
Matplotlib	3.8.0
Numba	0.55.2
NumPy	1.22.0
Pandas	2.0.3
PyMaxflow	1.3.0
SciPy	1.11.2
SimpleITK	2.2.1

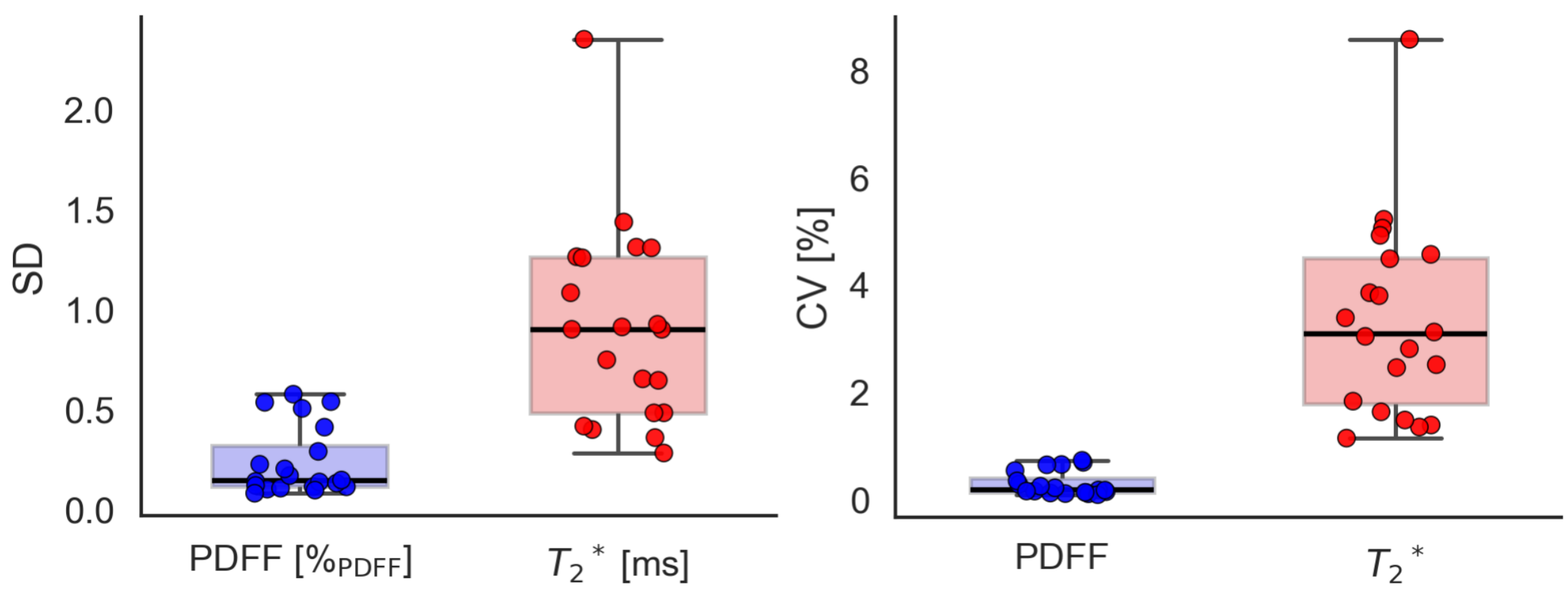

Figure S2: **Measurement variation**
The SD box plot on the left shows a low average PDFF spread consistent throughout all volunteers and an average $T_2{}^*$ SD below 1 ms. This results in CVs of below 4 % for both PDFF and $T_2{}^*$.

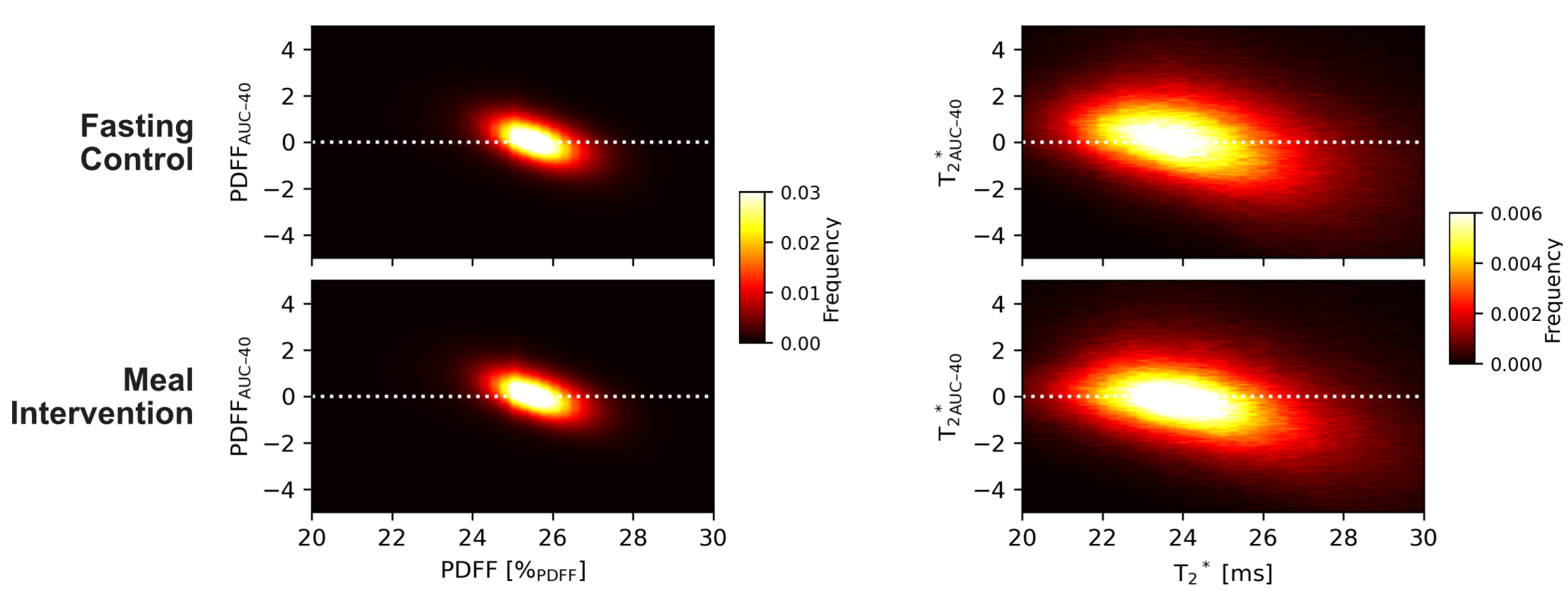

Figure S3: **Voxel-wise changes in muscle**
Both $\mathrm{PDFF}_{\mathrm{AUC}-40}$ and $T_2{}^*{}_{\mathrm{AUC}-40}$ of muscle did not alter from 0 for the majority of voxels. A bigger variation was observable for $T_2{}^*{}_{\mathrm{AUC}-40}$.

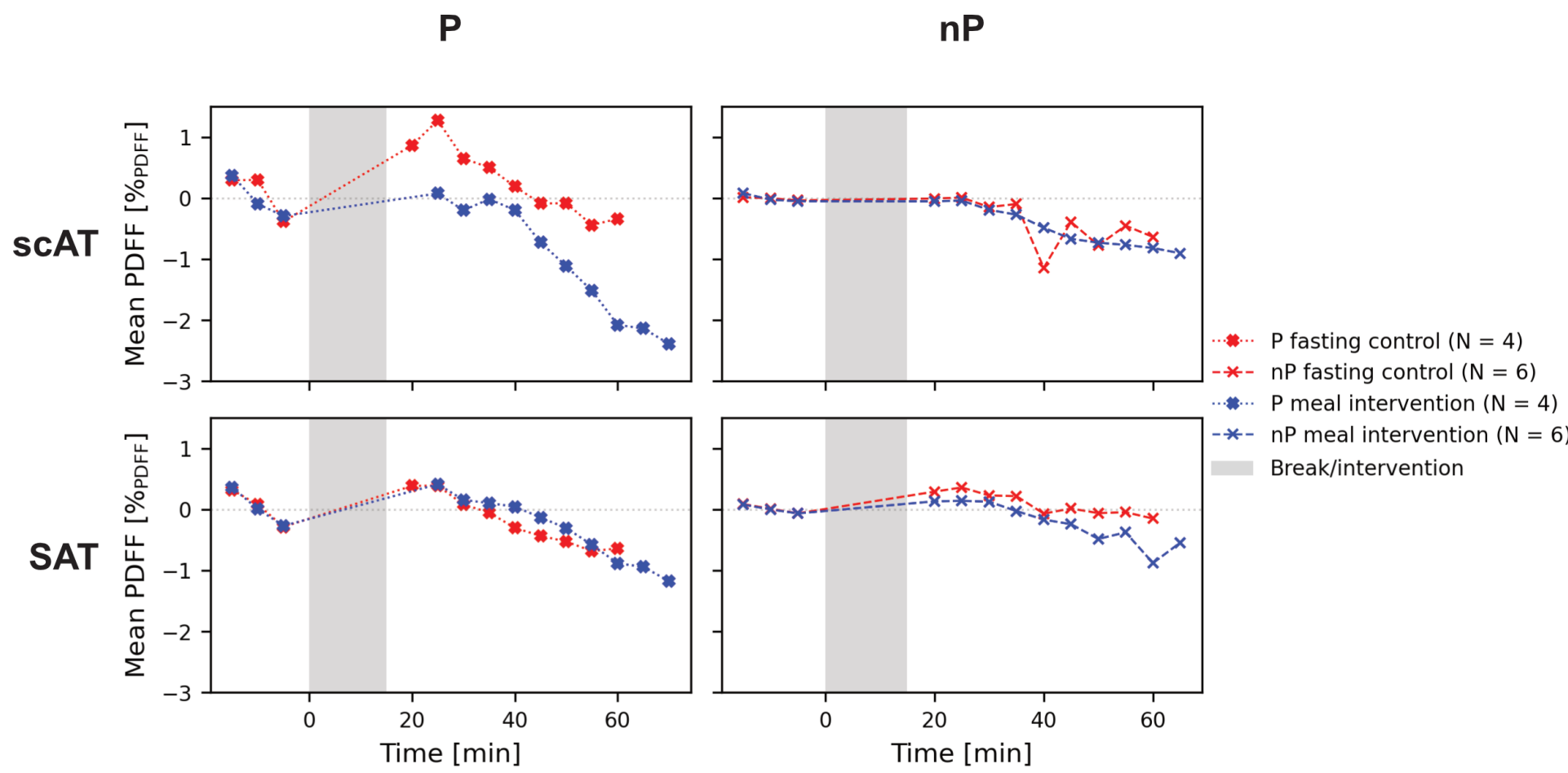

(a) Dynamic PDFF changes over time

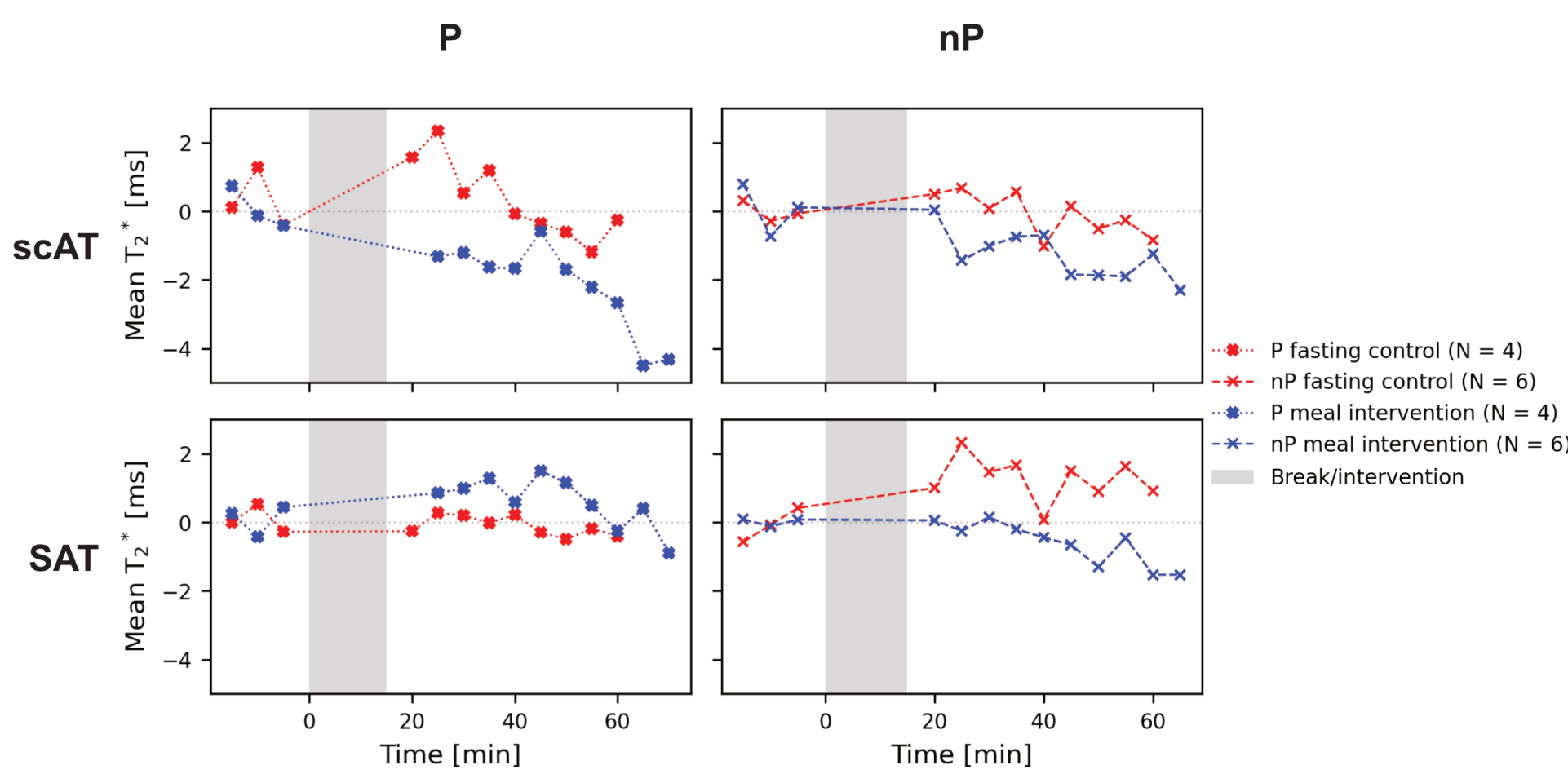

(b) Dynamic T_2^* changes over time

Figure S4: **Exploratory analysis of average dynamic mapping results: meal comparison**
(a) The dynamic PDFF changes in scAT in the top row and SAT in the bottom row are separated into the P group on the left and the nP group on the right. The red lines indicate dynamics from the fasting control, the blue lines dynamics from the meal intervention experiment and the gray area the time of break or intervention.
(b) The dynamics of $T_2{}^*$ are also displayed separated into the dynamics of the P group on the left and the nP group on the right for the scAT ROI in the top row and the SAT ROI in bottom row.

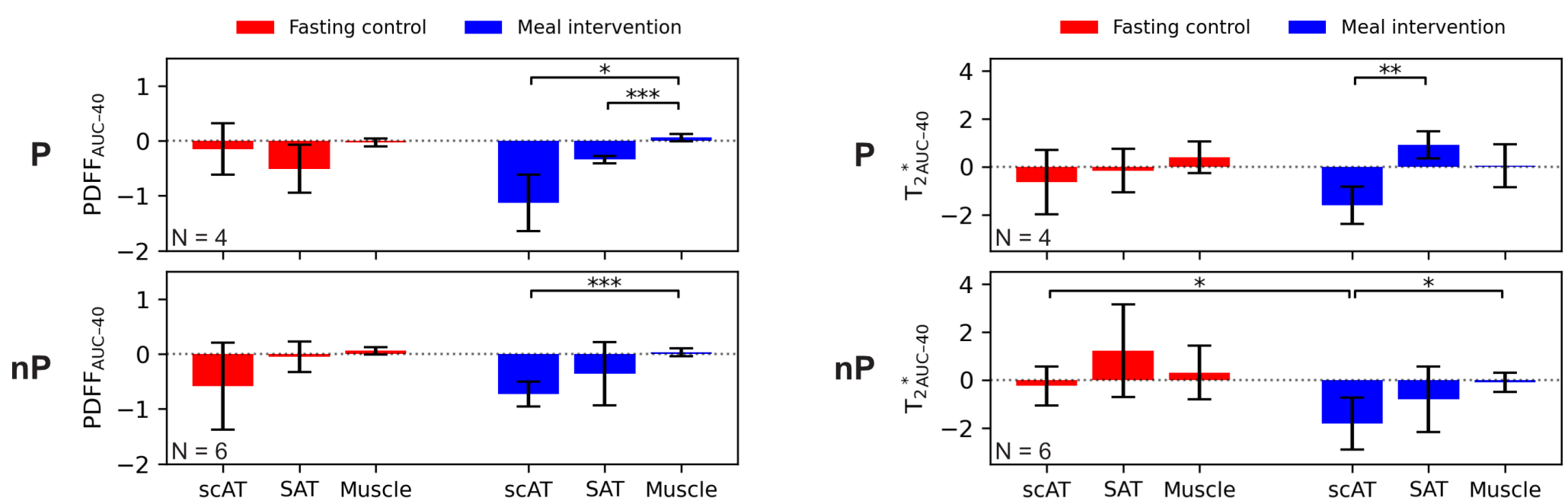

(a) Distribution of $\mathrm{PDFF}_{\mathrm{AUC-40}}$

(b) Distribution of ${T_2^*}_{\mathrm{AUC-40}}$

Figure S5: **Exploratory analysis of average dynamic mapping results: meal comparison**
(a) The mean and standard deviation of $\mathrm{PDFF}_{\mathrm{AUC-40}}$ is plotted for the P group at the top and the nP group at the bottom. Red bars indicate the fasting control experiments and blue bars the meal intervention experiments.
(b) The distribution of ${T_2{}^*}_{\mathrm{AUC-40}}$ is shown for the P group in the top plot and for the nP group in the bottom plot. Significant differences are indicated by asterisks.
*: $p < 0.05$, **: $p < 0.01$, ***: $p < 0.001$

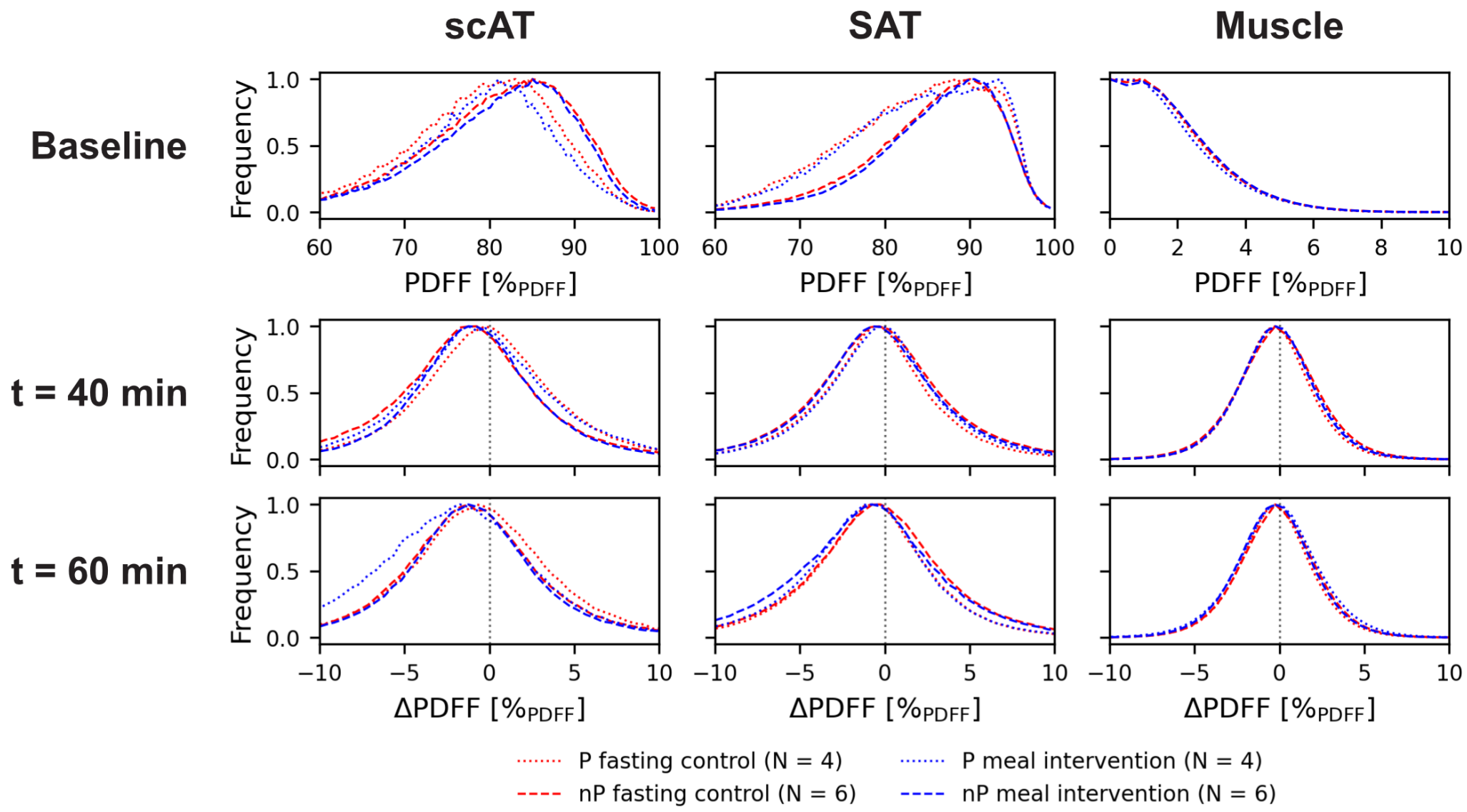

(a) PDFF histograms

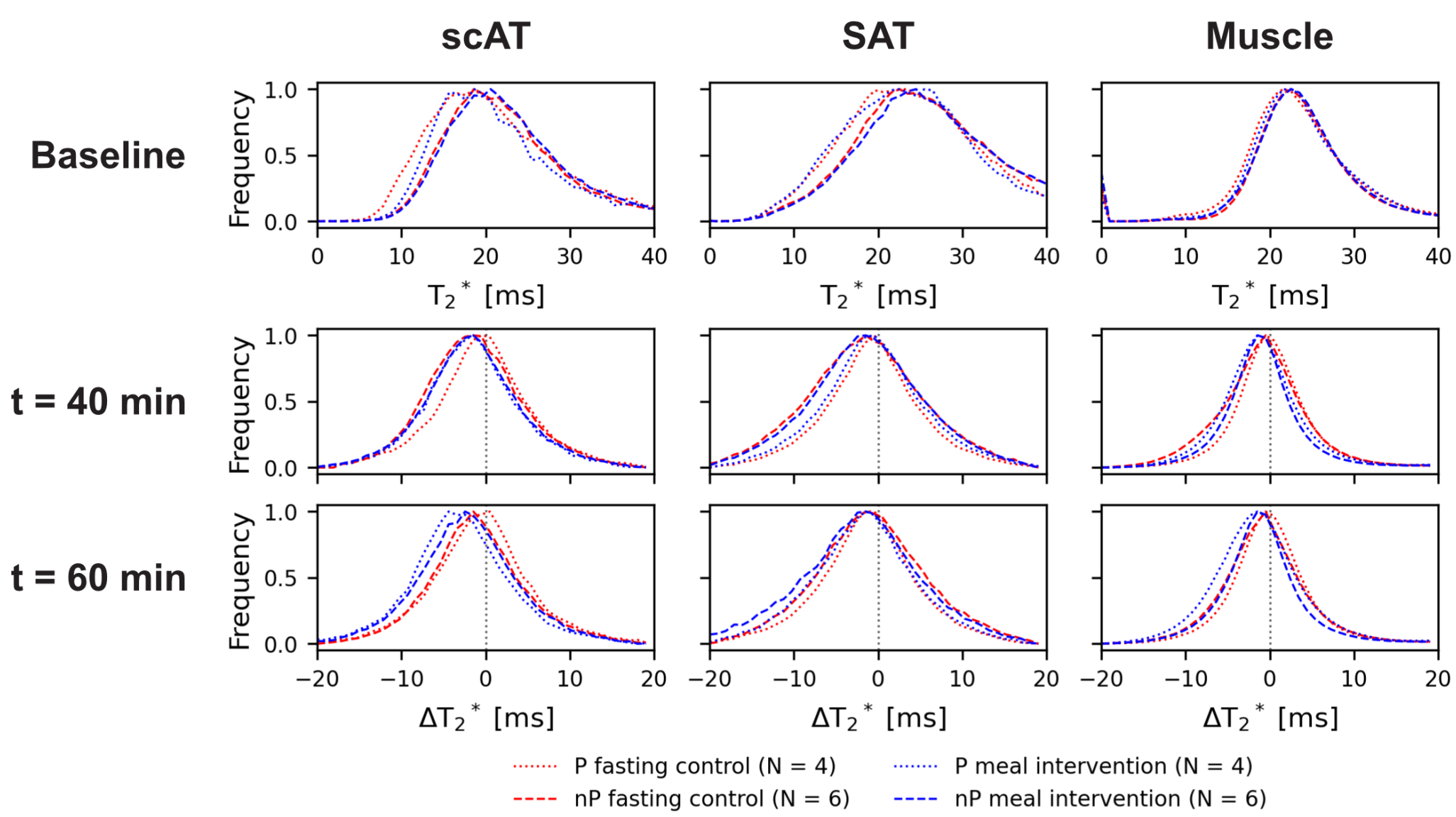

(b) T_2* histograms

Figure S6: **Average histograms: meal-comparison**
(a) At baseline, PDFF was lower in volunteers receiving the protein-rich food compared to other volunteers in scAT and SAT. In scAT, PDFF was decreasing slightly after $t = 40\,\text{min}$ in both groups but a stronger decrease was observable after $t = 60\,\text{min}$ in the P group upon activation.
(b) Similar to PDFF, there was also a difference of baseline T_2^* between the two groups. Strongest decrease of T_2^* was observed in the late dynamics in scAT of volunteers with the protein-rich food upon activation.
